# Peripheral level of CD33 and Alzheimer’s disease: a bidirectional two-sample Mendelian randomization study

**DOI:** 10.1038/s41398-022-02205-4

**Published:** 2022-10-03

**Authors:** Xiaojing Gu, Meng Dou, Bei Cao, Zheng Jiang, Yongping Chen

**Affiliations:** 1 Mental Health Center, West China Hospital, Sichuan University, Chengdu, Sichuan China; 2 Chengdu institute of computer application, Chinese Academy of Sciences, Chengdu, Sichuan China; 3 Department of Neurology, West China Hospital, Sichuan University, Chengdu, Sichuan China

**Keywords:** Psychiatric disorders, Genomics

## Abstract

Increased expression of CD33 in the brain has been suggested to be associated with increased amyloid plaque burden, while the peripheral level of CD33 in Alzheimer’s disease (AD) patients and its role in AD remain unclear. The current study aimed to systematically explore the bidirectional relationship between peripheral CD33 and AD. Genome-wide association study (GWAS) datasets of AD (N_cases_: 21982; N_controls_: 41944), blood CD33 mRNA level, the plasma CD33 protein level, and CD33 expression on immune-cell subtypes were obtained from GWASs conducted in the European population. Eligible IVs were extracted from the GWASs. MR estimates were calculated by inverse-variance weighting (IVW) and other sensitivity analyses. The main statistical analyses were conducted using TwoSampleMR (v.0.5.5) in R package (V.4.1.2).In the forward MR analysis (CD33 as exposure and AD as outcome), the IVW results indicated that elevated blood CD33 mRNA level (OR [95% CI] = 1.156[1.080, 1.238], *p* = 3.25e-05), elevated serum CD33 protein level (OR [95% CI] = 1.08 [1.031, 1.139], *p* = 1.6e-03) and increased CD33's expression on immune cell subtypes (*p* < 0.05) were all leading to a higher risk of AD. And sensitivity analyses supported these findings. While the reverse MR analysis (AD as exposure and CD33 as outcome) indicated that AD was not leading to the elevation of CD33's protein level in the blood (*p* > 0.05). In conclusion, our results indicated that elevated peripheral expression of CD33 was causal to the development of AD. Future studies are needed to work on developing CD33 as a biomarker and therapeutic target in AD.

## Introduction

Alzheimer’s disease (AD) is the most common neurodegenerative disorder in the elderly, which is clinically characterized by amnestic cognitive impairment and pathologically characterized by β-amyloid (Aβ)-containing extracellular plaques and tau-containing intracellular neurofibrillary tangles [1]. Aging is the most important risk factor for AD. Except for several causative genes such as *APP*, *PSEN1* and *PSEN2*, several risk genes for AD have also been identified by large genome-wide association analyses (GWASs), such as *APOE*, *TREM2*, *CD33* and *ABCA7* [2].

*CD33*, located on chromosome 19q13.3, is one of the top-ranked AD risk genes identified by GWAS and has been replicated in numerous genetic analyses [3]. CD33 belongs to the sialic acid-binding immunoglobulin (Ig)-like family and is a myeloid cell receptor, which is exclusively expressed by myeloid cells and microglia and participates in adhesion processes of human primary immune cells, mediating cell–cell interaction [4]. In AD, rs3865444 and rs12459419 are the two main CD33 single nucleotide polymorphisms (SNPs) that have been reported to be associated with the risk for AD [5]. Functional studies revealed that the protective allele of the rs3865444 was associated with a reduction in both CD33's expression and insoluble amyloid-beta 42 (A_42_) levels in AD brain [6]. Moreover, the mRNA level of CD33 in peripheral blood has also been found to be altered in AD patients, but the results remained controversial [7, 8].

Moreover, the underlying mechanisms for the elevation of CD33 in AD remain unclear. One of the most important questions was whether the altered level of CD33 in the blood is the cause or the result of AD? And whether the relationship between CD33 level and AD could be found at the mRNA level or the protein level? Moreover, since CD33 is a cell surface antigen, what kinds of cell subtypes are involved in the altered CD33 level? Because cell-specific studies could aid in identifying drug-targetable pathways and informing the design of precision treatments for diseases.

Mendelian randomization (MR) is a genetic method which applies genetic variants(SNPs) associated with the exposure as instrumental variables (IVs) in non-experimental design to assess the causal effect of the exposure on the outcome [9]. Compared to observational studies, the design of MR is able to avoid bias from unmeasured confounding factors and avoid bias from reverse causation [10]. Therefore, MR has been widely applied in identifying causal relationships between risk factors and diseases.

In the current study, we aimed to systematically explore the bidirectional relationship between CD33 and AD from blood CD33 mRNA level, the plasma CD33 protein level, and CD33's expression on immune-cell subtypes with a bi-directional 2-sample MR design.

## Methods

### GWAS Datasets

The expression of CD33 included 3 GWAS datasets: first is the blood CD33 mRNA level from the Expression quantitative trait loci (eQTL) analysis of gene expression, which investigated the genetics of blood gene expression by using eQTLGen Consortium data from 31,684 individuals [11]; second is the serum CD33 protein level from the genomic atlas of the human plasma proteome, which characterized the genetic architecture of the human plasma proteome in healthy blood donors from 3,301 individuals of European descent [12]; third is the expression of CD33 on immune cell subtypes from the GWAS on blood immune-cell-related trait, which assessed the impact of natural genetic variation on quantitative and discrete immune-related traits among 3,757 Sardinians [13].

For the GWAS of AD, we used the GWAS conducted by the International Genomics of Alzheimer’s Project (IGAP), which includes 21,982 clinically diagnosed late-onset AD(LOAD) cases and 41,944 cognitively normal controls to identify risk loci associated with AD [14] (Supplementary table 1).

### Identification of eligible IVs

MR is a genetic method which applies genetic variants associated with the exposure as IVs to make causal inferences of the exposure on the outcome [9]. Therefore, the most important and fundamental step of MR is to select eligible IVs. To identify genetic variants as eligible IVs, three key assumptions must be met: ﻿(1) the genetic variant should be directly associated with the exposure(relevance assumption); (2) the genetic variant should not be directly related to confounding factors(independence assumption); and (3) the genetic variant should not have a direct association with the outcome(exclusion assumption) [15]. Therefore, to meet assumption 1, on the one hand, we restricted the set of SNPs to be directly associated with the exposure at the genome-wide significant *p*-value threshold at *p* < 5e-08 as potential instruments; on the other hand, we removed the weak IVs judged by F-statistics, where a weak instrument was defined an F-statistic <10 [16]. Assumption 2 is calculated as horizontal pleiotropy, which can be calculated in the post-MR analysis. To meet assumption 3, we searched the PhenoScanner database [17] (a curated database of publicly available results from large-scale genetic association studies) for each IV to see whether they were directly associated with the outcome (*p* < 5e-08). And those IVs directly associated with the outcome should be removed.

### Bidirectional Two sample MR analysis

**Fig. 1:**
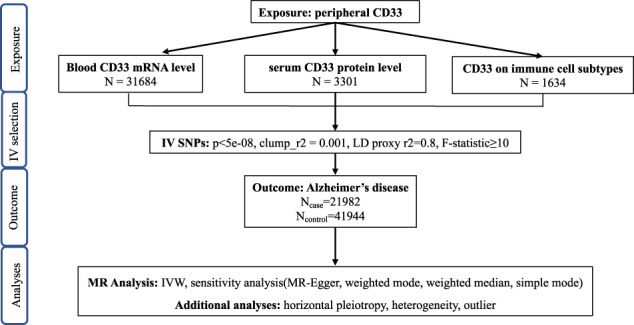
Flow diagram of the process for the bidirectional 2-sample Mendelian Randomization (MR) analysis. IV instrumental variables; SNP single nucleotide polymorphism.

Once the eligible IVs were selected, independent SNPs were clumped at a threshold of linkage disequilibrium LD at *r*^2^ = 0.001 within the window of 10 megabase pairs to avoid double counting and biased causal effect estimates. Next, the IVs were extracted from the outcome trait and were harmonized in both exposure and outcome GWAS. In this step, palindromic SNPs with intermediate allele frequency were removed. Moreover, if a particular requested SNP is not present in the outcome GWAS, then an SNP (proxy) that is in LD with the requested SNP (target) will be searched, which was defined using 1000 genomes European sample data (*r*^2^ ≥ 0.8). Once the exposure and outcome data are harmonized, MR can be performed. The Wald ratio test was used to calculate the causative effect of the exposure on the outcome when a single IV is available, while the inverse variance weighted (IVW) method was performed as the main analysis when multiple IVs were available [18], which is the most efficient analysis method with valid IVs because it accounts for heterogeneity in the variant-specific causal estimates [19]. Moreover, additional sensitivity analyses including the simple mode, weighted mode, weighted median and MR-Egger regression methods, were further conducted to assess the robustness of the findings [19]. And we used the MR Egger intercept, Cochran Q statistic and MR-PRESSO global test to test the presence of directional pleiotropy, IV heterogeneity and outlier IV, respectively [20]. The main statistical analyses were conducted using TwoSampleMR (v.0.5.5) in the R package(V.4.1.2) [15]. The flowchart of the study was presented in Fig. 1.

## Results

**Fig. 2:**
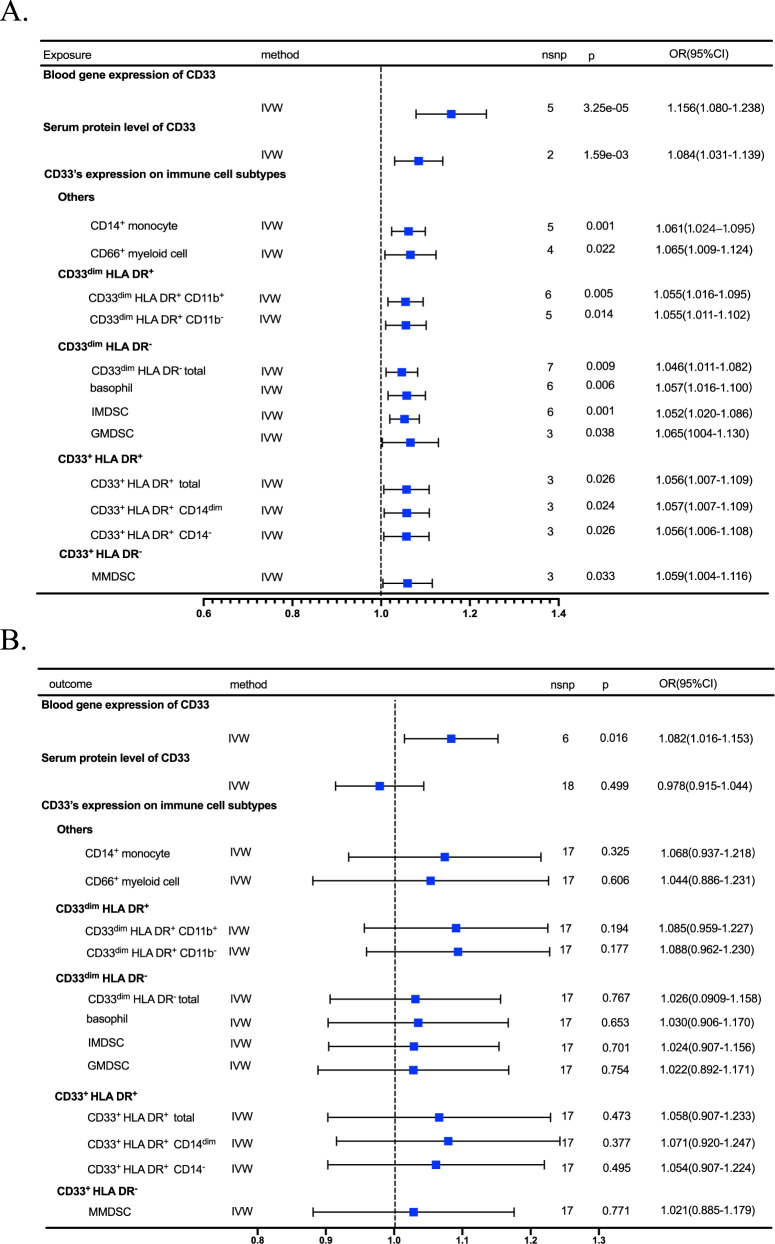
Forest plot for the IVW results of bidirectional 2-sample MR analyses. **A** AD as the outcome, and CD33 traits as exposure, forest plot showing the causal effect of CD33 on AD; **B** AD as exposure, and CD33 traits as the outcome, forest plot showing the causal effect of AD on CD33.

In the forward MR analysis (CD33 as exposure and AD as outcome), we analyzed the causal effect of the blood CD33 level on AD. The eligible IVs were not directly associated with the outcome (Supplementary Table 2 and Supplementary Table 3). In the blood CD33 mRNA level, 5 IVs were available, and the IVW results indicated that each one standard deviation increase in blood CD33 gene expression was leading to a higher risk of AD (OR [95% CI] = 1.156 [1.080, 1.238], *p* = 3.25e-05), and such results were supported with another 3 MR methods, including weighted median (OR [95% CI] = 1.162[1.080, 1.250], *p* = 5.08e-05), simple mode (OR [95% CI] = 1.211[1.071, 1.370], *p* = 0.038), and weighted mode (OR [95% CI] = 1.165[1.088, 1.249], *p* = 0.012). And the MR-Egger method showed a marginal association and suggested the same direction of effect (OR [95% CI] = 1.206[1.066, 1.365], *p* = 0.059). Next, at the serum CD33 protein level, 2 IVs were eligible for the MR analysis, and the IVW results showed that﻿ each one standard deviation increase in serum CD33 protein level was also leading to an increased risk of AD (OR [95% CI] = 1.08 [1.031, 1.139], *p* = 1.6e-03). Lastly, in the analysis of CD33's expression on immune cell subtypes, the MR results showed that increased expression on all CD33 + cell subtypes, including CD14^+^ monocytes, CD66b^++^ myeloid cells, CD33^dim^ cells and CD33^+^ cells were all leading to a higher risk of AD (*p* < 0.05) with IVW and other sensitivity analyses (Fig. 2A and Supplementary Table 4).

In the reverse MR analysis (AD as exposure and CD33 as outcome), we studied the causal effect of AD on blood CD33 levels. The eligible IVs were not directly associated with the outcome (Supplementary Table 5 and Supplementary Table 6). Regarding the blood CD33 mRNA level, the IVW result indicated that AD was leading to the elevated CD33 mRNA level in the blood (OR [95% CI] = 1.082[1.016, 1.153], *p* = 0.016), but the result was not supported by other sensitivity analyses(*p* > 0.05). Moreover, the MR results also indicated that AD was not leading to the elevation of either the serum CD33 protein level or CD33's expression on immune cell subtypes (*p* > 0.05) (Fig. 2B and Supplementary Table 4).

Next, we performed extensive analyses to validate the causal association between CD33 and AD. The Cochran’s Q test indicated some heterogeneity among the IVs (Table 1). Nonetheless, the intercept of MR-Egger is not significantly deviated from zero, suggesting no apparent horizontal pleiotropy, and the MR-PRESSO analysis detected no potential instrumental outlier at the nominal significance level of 0.05 (Table 1).

**Table 1:** Results for the horizontal pleiotropy, heterogeneity and outlier test.

		pleiotropy			Heterogeneity						MR-PRESSO
		egger_intercept	se	pval	MR-Egger			IVW			
					Q	Q_df	Q_pval	Q	Q_df	Q_pval	
AD as outcome	Exposure										
	Blood gene expression of CD33	−0.011	0.014	0.481	2.025	3	0.567	2.669	4	0.615	0.674
	Serum protein level of CD33	NA	NA	NA	NA	NA	NA	2.357	1	0.125	NA
	CD33 on CD14 + monocyte	−0.016	0.02	0.497	6.771	3	0.08	8.109	4	0.088	0.381
	CD33 on CD66b + + myeloid cell	−0.044	0.015	0.101	1.716	2	0.423	10.111	3	0.018	0.369
	CD33 on CD33dim HLA DR + CD11b +	−0.025	0.015	0.182	7.285	4	0.122	12.016	5	0.035	0.312
	CD33 on CD33dim HLA DR + CD11b-	−0.024	0.018	0.274	7.643	3	0.054	12.197	4	0.016	0.246
	CD33 on CD33dim HLA DR-	−0.044	0.024	0.143	7.105	4	0.13	12.991	5	0.023	0.308
	CD33 on basophil	−0.054	0.022	0.058	5.077	5	0.406	11.191	6	0.083	0.39
	CD33 on IMDSC	−0.047	0.022	0.111	3.317	4	0.506	7.476	5	0.187	0.422
	CD33 on GMDSC	−0.04	0.026	0.37	1.774	1	0.182	5.89	2	0.052	NA
	CD33 on CD33 + HLA DR +	−0.047	0.017	0.226	0.772	1	0.379	8.012	2	0.018	0.372
	CD33 on CD33 + HLA DR + CD14dim	−0.458	0.017	0.228	0.741	1	0.389	7.844	2	0.02	0.4
	CD33 on CD33 + HLA DR + CD14-	−0.047	0.017	0.226	0.806	1	0.369	8.046	2	0.018	0.397
	CD33 on MMDSC	−0.47	0.02	0.261	1.359	1	0.243	8.508	2	0.014	0.404
AD as exposure	Outcome										
	Blood gene expression of CD33	0.006	0.012	0.651	2.419	4	0.659	2.657	5	0.753	0.734
	Serum protein level of CD33	0.006	0.010	0.580	10.940	16	0.813	11.259	17	0.843	0.681
	CD33 on CD14 + monocyte	−0.024	0.019	0.227	17.964	15	0.265	19.866	16	0.226	0.273
	CD33 on CD66b + + myeloid cell	−0.006	0.025	0.803	27.424	15	0.025	27.542	16	0.036	0.095
	CD33 on CD33dim HLA DR + CD11b +	−0.020	0.018	0.289	16.398	15	0.356	17.718	16	0.341	0.376
	CD33 on CD33dim HLA DR + CD11b-	−0.019	0.018	0.327	16.505	15	0.349	17.636	16	0.346	0.376
	CD33 on CD33dim HLA DR-	−0.020	0.019	0.307	16.561	15	0.346	17.795	16	0.336	0.452
	CD33 on basophil	−0.024	0.018	0.214	14.342	15	0.500	16.028	16	0.451	0.543
	CD33 on IMDSC	−0.014	0.018	0.458	15.188	15	0.438	15.775	16	0.469	0.56
	CD33 on GMDSC	−0.016	0.020	0.444	11.634	15	0.707	12.253	16	0.726	0.848
	CD33 on CD33 + HLA DR +	−0.022	0.023	0.346	24.195	15	0.062	25.724	16	0.058	0.091
	CD33 on CD33 + HLA DR + CD14dim	−0.025	0.023	0.293	23.254	15	0.079	25.096	16	0.068	0.088
	CD33 on CD33 + HLA DR + CD14-	−0.021	0.023	0.371	23.156	15	0.081	24.470	16	0.080	0.117
	CD33 on MMDSC	−0.039	0.020	0.068	17.746	15	0.276	22.318	16	0.133	0.156

## Discussion

Our results systematically investigated the bidirectional relationship between CD33 and AD from blood CD33 mRNA level, serum CD33 protein level, and expression of CD33 on specific immune cell subtypes. Our results indicated that elevated peripheral expression of CD33 was linked to the development of AD, while AD might not be the cause for CD33's elevation in the blood.

In performing MR, the most important and fundamental step is that a genetic variant must be a valid IV. To achieve this, three key assumptions should be met [15]. Assumption 1 is that the IV should be associated with the exposure. In our study, we have selected the IVs from large GWAS datasets and we selected the SNPs significantly associated with exposures which passed the stringent GWAS threshold at *p* < 5e-08. Furthermore, we avoided weak IVs based on the F-statistic < 10 [16]. After these steps, assumption 1 was met. Assumption 2 was calculated as horizontal pleiotropy in the current study, and our results showed no horizontal pleiotropy effect in our analyses(p > 0.05). Therefore, assumption 2 was met. Assumption 3 is that IVs should not be directly associated with the outcome. To meet assumption 3, ﻿we searched the PhenoScanner database [17] for each IV to see whether they were directly associated with the outcome (*p* < 5e-08).As a result, no IV was found to be directly associated with the outcome. Therefore, assumption 3 was met. These results indicated that the IVs used in the current study were strong, which ensured our MR results were valid.

CD33 has been widely studied in AD. Previous studies have found that higher CD33 expression in the parietal lobe is associated with more advanced cognitive decline or disease status [21], and knocking out CD33 results in lower Aβ levels and reduced amyloid plaque burden in the brain [6]. Several independent GWASs have also identified CD33 as a strong genetic locus linked to late-onset AD (LOAD), where rs3865444 and rs12459419 were the most commonly studied SNPs [5]. Previous studies have revealed that the protective allele of the rs3865444 was located in the promotor region of CD33, and the protective allele was associated with a reduction in both CD33 expression and insoluble Aβ42 levels in AD brain, especially in the microglial cells [6]. Further functional studies found that CD33 inhibited uptake and clearance of Aβ42 in cell and animal models of AD [6]. Rs12459419 was located in exon 2 and was in linkage equilibrium with rs3865444 [22]. The protective allele of rs12459419 “T” enhances exon skipping and leads to the increased production of a short isoform of CD33, known as human CD33m [22]. A recent study in cell and animal models has found that compared to the wild type of human CD33(human CD33M), human CD33m is a gain-of-function variant, which enhances Aβ_1–42_ phagocytosis in microglia [23].

The peripheral level of CD33 has also been studied. Heidari et al’s study compared CD33 mRNAs expression on leucocytes between 233 LOAD patients and 238 controls, which found a significant increase in CD33 mRNA expression levels in white blood cells of LOAD patients [7]. However, another previous study found that expression of CD33 mRNA in peripheral blood mononuclear cells was down-regulated in AD patients compared to controls and the frequency of CD33 positive monocytes was also lower in AD patients than in controls [8]. Moreover, a recent study which used a different GWAS dataset of AD and a different dataset of serum proteome dataset with two-sample MR found a significant causal association between serum CD33 and AD [24], which is consistent with our results. Besides, the study also ﻿conducted reverse MR analysis (using AD as the exposure and serum CD33 as the outcome) and confirmed that AD is causal for an increased CD33 protein level [24]. However, in our study, we only found that AD was causal for an increased CD33 mRNA level by the IVW method, which was not supported by other sensitivity analyses; moreover, we also failed to identify the causal effect of AD on serum CD33 protein level. We infer that the discrepancy between our results and the previous results was caused by the utilization of different exposure and outcome datasets: the serum CD33 protein association in their study was obtained from 2893 samples from two Greek population-based cohorts [24]; and the AD GWAS dataset used in their study was from a previous AD GWAS conducted in 2013 [25]. Therefore, more studies are warranted to specify the causal effect of AD on the serum CD33 level.

In brief, our study found that both mRNA level and protein level of CD33 in the blood was causal for AD, which partially supports that elevation of CD33 protein in the serum is caused by upregulated gene expression but not impaired protein degradation. These results provide further insights into the promising application of CD33 in AD. Firstly, the peripheral level of CD33 could be served as a biomarker for diagnosing AD and monitoring disease progression. Moreover, CD33 might be applied as a promising therapeutic target for AD, including anti-CD33 antibodies and small molecules targeting CD33.

## Conclusion

In conclusion, our results indicated that elevated peripheral expression of CD33 was linked to the development of AD. Future studies are needed to work on developing CD33 as a biomarker and therapeutic target in AD.

## Supplementary information

supplementary tables

## Data Availability

The original datasets of the current study are available in the ieu open gwas project(https://gwas.mrcieu.ac.uk/). The datasets analyzed during the current study are available from the corresponding author upon reasonable request.

## Code availability

The main statistical analyses were conducted using TwoSampleMR (v.0.5.5) in the R package (V.4.1.2).
