# Supplementary material for: Peripheral level of CD33 and Alzheimer’s disease: a bidirectional two-sample Mendelian randomization study
Source: Transl Psychiatry. 2022 Oct 3;12:427. doi: 10.1038/s41398-022-02205-4 (PMC9529877; doi:10.1038/s41398-022-02205-4)
Supplement: Supplementary file 1 — supplementary tables [file 41398_2022_2205_MOESM1_ESM.pdf]

Supplementary table 1. Description of the consortium data sets used for AD GWAS.

	Alzheimer's disease cases			Controls		
**Consortium**	***N***	**Percent female**	**Mean AAO (s.d)**	***N***	**Percent female**	**Mean AAE (s.d)**
ADGC	14,428	59.3	71.1 (17.3)	14,562	59.3	76.2 (9.9)
CHARGE	2,137	67.3	82.6 (12)	13,474	55.8	76.7 (8.2)
EADI	2,240	65	75.4 (9.1)	6,631	60.6	78.9 (7.0)
GERAD	3,177	64	73.0 (0.2)	7,277	51.8	51.0 (0.1)
N	21,982			41,944		

Supplementary table 2: IVs used in the forward MR analyses (CD33 as exposure, AD as outcome).

exposure || id	Sample.size	se	pval	beta	pos	SNP	EA	OA	eaf
CD33 on CD14+ monocyte || id:ebi-a-GCST90001946	1634	0.03378	1.05E-190	-1.143	19:51727962	rs3865444	A	C	0.2121
CD33 on CD14+ monocyte || id:ebi-a-GCST90001946	1634	0.03612	6.13E-09	-0.2111	19:52254351	rs4801891	C	T	0.563
CD33 on CD14+ monocyte || id:ebi-a-GCST90001946	1634	0.03593	6.32E-15	0.2828	19:51591138	rs78949787	T	C	0.5535
CD33 on CD14+ monocyte || id:ebi-a-GCST90001946	1634	0.06809	3.79E-12	-0.4764	19:52897142	rs11882720	G	T	0.0731
CD33 on CD14+ monocyte || id:ebi-a-GCST90001946	1634	0.04013	7.97E-23	-0.4007	19:51574038	rs7351079	G	A	0.2708
CD33 on CD14+ monocyte || id:ebi-a-GCST90001946	1634	0.07202	1.39E-09	0.4387	19:51868443	rs58483275	T	C	0.0704

CD33 on CD33+ HLA DR+ CD14dim || id:ebi-a-GCST90001947	1577	0.06754	3.29E-10	-0.4272	19:52097261	rs58301181	G	C	0.0843
CD33 on CD33+ HLA DR+ CD14dim || id:ebi-a-GCST90001947	1577	0.04234	2.43E-14	-0.326	19:51574038	rs7351079	G	A	0.2613
CD33 on CD33+ HLA DR+ CD14dim || id:ebi-a-GCST90001947	1577	0.03704	1.35E-14	0.288	19:51588018	rs62114147	T	C	0.5568
CD33 on CD33+ HLA DR+ CD14dim || id:ebi-a-GCST90001947	1577	0.03655	7.83E-184	-1.214	19:51727962	rs3865444	A	C	0.1934
CD33 on CD33dim HLA DR+ CD11b+ || id:ebi-a-GCST90001948	1633	0.0387	3.92E-11	-0.2575	1:161479745	rs1801274	G	A	0.3298
CD33 on CD33dim HLA DR+ CD11b+ || id:ebi-a-GCST90001948	1633	0.03571	4.04E-16	0.2936	19:51588018	rs62114147	T	C	0.5508
CD33 on CD33dim HLA DR+ CD11b+ || id:ebi-a-GCST90001948	1633	0.04	8.18E-22	-0.3895	19:51574038	rs7351079	G	A	0.271
CD33 on CD33dim HLA DR+ CD11b+ || id:ebi-a-GCST90001948	1633	0.07133	6.18E-12	0.4941	19:51868443	rs58483275	T	C	0.0704
CD33 on CD33dim HLA DR+ CD11b+ || id:ebi-a-GCST90001948	1633	0.03595	7.66E-10	-0.2225	19:52254351	rs4801891	C	T	0.5628
CD33 on CD33dim HLA DR+ CD11b+ || id:ebi-a-GCST90001948	1633	0.03358	2.38E-191	-1.139	19:51727962	rs3865444	A	C	0.2122
CD33 on CD33dim HLA DR+ CD11b+ || id:ebi-a-GCST90001948	1633	0.06841	7.43E-11	-0.4484	19:52897142	rs11882720	G	T	0.0726
CD33 on CD33dim HLA DR+ CD11b- || id:ebi-a-GCST90001949	1634	0.03857	1.84E-11	-0.261	1:161479745	rs1801274	G	A	0.3299
CD33 on CD33dim HLA DR+ CD11b- || id:ebi-a-GCST90001949	1634	0.03992	2.91E-21	-0.3832	19:51574038	rs7351079	G	A	0.2708

CD33 on CD33dim HLA DR+ CD11b- || id:ebi-a-GCST90001949	1634	0.03559	1.24E-16	0.2978	19:51588018	rs62114147	T	C	0.5511
CD33 on CD33dim HLA DR+ CD11b- || id:ebi-a-GCST90001949	1634	0.03392	2.40E-183	-1.119	19:51727962	rs3865444	A	C	0.2121
CD33 on CD33dim HLA DR+ CD11b- || id:ebi-a-GCST90001949	1634	0.0359	1.13E-08	-0.2061	19:52254351	rs4801891	C	T	0.563
CD33 on CD33dim HLA DR+ CD11b- || id:ebi-a-GCST90001949	1634	0.105	7.88E-09	-0.6094	19:48582916	rs73051934	G	A	0.0278
CD33 on CD33dim HLA DR+ CD11b- || id:ebi-a-GCST90001949	1634	0.06357	1.81E-10	-0.4079	19:52097261	rs58301181	G	C	0.0872
CD33 on Granulocytic Myeloid-Derived Suppressor Cells || id:ebi-a-GCST90001950	1244	0.04091	3.22E-08	0.2277	19:51591138	rs78949787	T	C	0.5691
CD33 on Granulocytic Myeloid-Derived Suppressor Cells || id:ebi-a-GCST90001950	1244	0.04903	1.02E-58	-0.8358	19:51731176	rs7245846	A	G	0.1853
CD33 on Granulocytic Myeloid-Derived Suppressor Cells || id:ebi-a-GCST90001950	1244	0.048	5.38E-09	-0.2821	19:51574038	rs7351079	G	A	0.2544
CD33 on CD66b++ myeloid cell || id:ebi-a-GCST90001951	1464	0.04205	1.74E-10	0.2704	19:52161035	rs12459503	C	T	0.7148
CD33 on CD66b++ myeloid cell || id:ebi-a-GCST90001951	1464	0.03799	2.62E-12	0.2681	19:51591138	rs78949787	T	C	0.5574
CD33 on CD66b++ myeloid cell || id:ebi-a-GCST90001951	1464	0.04316	1.04E-12	-0.3103	19:51574038	rs7351079	G	A	0.2702
CD33 on CD66b++ myeloid cell ||	1464	0.0394	2.39E-108	-0.9497	19:51727962	rs3865444	A	C	0.2094

id:ebi-a-GCST90001951									
CD33 on Monocytic Myeloid-Derived Suppressor Cells || id:ebi-a-GCST90001952	1569	0.03884	1.71E-153	-1.151	19:51727962	rs3865444	A	C	0.1896
CD33 on Monocytic Myeloid-Derived Suppressor Cells || id:ebi-a-GCST90001952	1569	0.06721	1.94E-08	-0.3795	19:52097261	rs58301181	G	C	0.0848
CD33 on Monocytic Myeloid-Derived Suppressor Cells || id:ebi-a-GCST90001952	1569	0.03721	2.56E-12	0.2625	19:51588018	rs62114147	T	C	0.5586
CD33 on Monocytic Myeloid-Derived Suppressor Cells || id:ebi-a-GCST90001952	1569	0.04232	9.03E-18	-0.3677	19:51574038	rs7351079	G	A	0.2604
CD33 on CD33dim HLA DR- || id:ebi-a-GCST90001953	1558	0.0784	2.26E-10	0.5006	1:247719769	rs56043070	A	G	0.0565
CD33 on CD33dim HLA DR- || id:ebi-a-GCST90001953	1558	0.0392	4.22E-11	-0.2604	19:53262327	rs11669394	A	G	0.3039
CD33 on CD33dim HLA DR- || id:ebi-a-GCST90001953	1558	0.03974	2.60E-145	-1.138	19:51727962	rs3865444	A	C	0.1845
CD33 on CD33dim HLA DR- || id:ebi-a-GCST90001953	1558	0.07038	4.12E-12	-0.4918	19:51742145	rs75773078	A	G	0.0709
CD33 on CD33dim HLA DR- || id:ebi-a-GCST90001953	1558	0.04579	1.22E-13	0.3426	19:51544665	rs138712525	A	G	0.199
CD33 on CD33dim HLA DR- || id:ebi-a-GCST90001953	1558	0.04209	1.15E-17	-0.3645	19:51574038	rs7351079	G	A	0.258
CD33 on basophil ||	1555	0.07845	6.30E-10	0.4881	1:247719769	rs56043070	A	G	0.0566

id:ebi-a-GCST90001954									
CD33 on basophil || id:ebi-a-GCST90001954	1555	0.04576	1.26E-13	0.3421	19:51538952	rs79377141	A	G	0.2006
CD33 on basophil || id:ebi-a-GCST90001954	1555	0.07051	4.53E-12	-0.4918	19:51742145	rs75773078	A	G	0.0707
CD33 on basophil || id:ebi-a-GCST90001954	1555	0.04217	1.40E-17	-0.3642	19:51574038	rs7351079	G	A	0.2579
CD33 on basophil || id:ebi-a-GCST90001954	1555	0.07238	1.33E-12	0.5176	19:51868443	rs58483275	T	C	0.0733
CD33 on basophil || id:ebi-a-GCST90001954	1555	0.03861	1.81E-150	-1.131	19:51731176	rs7245846	A	G	0.1939
CD33 on basophil || id:ebi-a-GCST90001954	1555	0.06799	2.04E-15	-0.5454	19:51897760	rs111695695	A	C	0.0772
CD33 on Immature Myeloid-Derived Suppressor Cells || id:ebi-a-GCST90001955	1554	0.0393	6.08E-143	-1.115	19:51731176	rs7245846	A	G	0.1934
CD33 on Immature Myeloid-Derived Suppressor Cells || id:ebi-a-GCST90001955	1554	0.07057	1.64E-13	-0.5252	19:51742145	rs75773078	A	G	0.0705
CD33 on Immature Myeloid-Derived Suppressor Cells || id:ebi-a-GCST90001955	1554	0.0459	3.07E-13	0.3376	19:51538952	rs79377141	A	G	0.2008
CD33 on Immature Myeloid-Derived Suppressor Cells || id:ebi-a-GCST90001955	1554	0.0424	5.07E-15	-0.3351	19:51574038	rs7351079	G	A	0.258

CD33 on Immature Myeloid-Derived Suppressor Cells || id:ebi-a-GCST90001955	1554	0.06836	1.06E-13	-0.5128	19:51897760	rs111695695	A	C	0.0772
CD33 on Immature Myeloid-Derived Suppressor Cells || id:ebi-a-GCST90001955	1554	0.07256	5.21E-12	0.5046	19:51868443	rs58483275	T	C	0.0734
CD33 on CD33+ HLA DR+ || id:ebi-a-GCST90001956	1579	0.03627	2.81E-187	-1.219	19:51727962	rs3865444	A	C	0.1938
CD33 on CD33+ HLA DR+ || id:ebi-a-GCST90001956	1579	0.06738	1.94E-10	-0.4318	19:52097261	rs58301181	G	C	0.0842
CD33 on CD33+ HLA DR+ || id:ebi-a-GCST90001956	1579	0.03693	3.04E-15	0.2943	19:51588018	rs62114147	T	C	0.5567
CD33 on CD33+ HLA DR+ || id:ebi-a-GCST90001956	1579	0.04212	2.54E-15	-0.3366	19:51574038	rs7351079	G	A	0.2619
CD33 on CD33+ HLA DR+ CD14- || id:ebi-a-GCST90001957	1578	0.03698	3.26E-15	0.2943	19:51588018	rs62114147	T	C	0.557
CD33 on CD33+ HLA DR+ CD14- || id:ebi-a-GCST90001957	1578	0.04213	1.07E-15	-0.3413	19:51574038	rs7351079	G	A	0.2617
CD33 on CD33+ HLA DR+ CD14- || id:ebi-a-GCST90001957	1578	0.0674	6.49E-11	-0.4433	19:52097261	rs58301181	G	C	0.0843
CD33 on CD33+ HLA DR+ CD14- || id:ebi-a-GCST90001957	1578	0.0362	9.18E-189	-1.223	19:51727962	rs3865444	A	C	0.1936
ENSG00000105383 || id:eqtl-a-ENSG00000105383	30077	0.0165533	1.02E-49	-0.245385	7:50370254	rs149007767	T	C	0.149708
ENSG00000105383 || id:eqtl-a-ENSG00000105383	31569	0.0120238	9.85E-10	-0.0734873	8:130613614	rs10098310	A	G	0.575007

ENSG00000105383 || id:eqtl-a-ENSG00000105383	31569	0.0118845	4.69E-16	0.0964906	14:23589349	rs2239630	G	A	0.519173
ENSG00000105383 || id:eqtl-a-ENSG00000105383	25419	0.0413475	2.47E-130	-1.00435	19:51761614	rs62115999	A	G	0.0203008
ENSG00000105383 || id:eqtl-a-ENSG00000105383	16223	0.0461519	4.29E-11	0.304316	19:51739215	rs147493755	C	T	0.016864
ENSG00000105383 || id:eqtl-a-ENSG00000105383	31569	0.0116707	1.00E-200	0.363274	19:51726911	rs1710398	C	A	0.418566
Myeloid cell surface antigen CD33 || id:prot-a-439	3301	0.0424	4.68E-09	-0.2485	19:51575009	rs62114140	T	C	0.10014
Myeloid cell surface antigen CD33 || id:prot-a-439	3301	0.0209	1.00E-200	-0.9436	19:51728477	rs12459419	T	C	0.32917

EA: effect allele; OA: other allele; eaf: allele frequency of effect allele.

Supplementary table 3: Phenoscanncer search results of the IVs used in the forward MR analyses(CD33 as exposure, AD as outcome).

snp	position	trait	study	pmid	ancestry
rs3865444	chr19:51727962	Eosinophil count	Astle W	27863252	European
rs3865444	chr19:51727962	Granulocyte count	Astle W	27863252	European
rs3865444	chr19:51727962	Lymphocyte count	Astle W	27863252	European
rs3865444	chr19:51727962	Monocyte count	Astle W	27863252	European
rs3865444	chr19:51727962	Myeloid white cell count	Astle W	27863252	European
rs3865444	chr19:51727962	Platelet count	Astle W	27863252	European
rs3865444	chr19:51727962	Plateletcrit	Astle W	27863252	European
rs3865444	chr19:51727962	Sum eosinophil basophil counts	Astle W	27863252	European
rs3865444	chr19:51727962	Sum neutrophil eosinophil counts	Astle W	27863252	European
rs3865444	chr19:51727962	White blood cell count	Astle W	27863252	European

rs3865444	chr19:51727962	Alzheimers disease	Hollingworth P	21460840	European
rs3865444	chr19:51727962	Late onset Alzheimers disease	Naj AC	21460841	European
rs3865444	chr19:51727962	Alzheimers disease	IGAP	24162737	European
rs3865444	chr19:51727962	Alzheimers disease late onset	Naj AC	21460841	European
rs3865444	chr19:51727962	Alzheimers disease late onset	IGAP	24162737	European
rs3865444	chr19:51727962	Platelet count	Astle W	27863252	European
rs3865444	chr19:51727962	White blood cell count	Astle W	27863252	European
rs3865444	chr19:51727962	Alzheimer disease	Naj AC	21460841	European
rs58483275	chr19:51868443	Myositis	Neale B	UKBB	European
rs3865444	chr19:51727962	Eosinophil count	Astle W	27863252	European
rs3865444	chr19:51727962	Granulocyte count	Astle W	27863252	European
rs3865444	chr19:51727962	Lymphocyte count	Astle W	27863252	European
rs3865444	chr19:51727962	Monocyte count	Astle W	27863252	European
rs3865444	chr19:51727962	Myeloid white cell count	Astle W	27863252	European
rs3865444	chr19:51727962	Platelet count	Astle W	27863252	European
rs3865444	chr19:51727962	Plateletcrit	Astle W	27863252	European
rs3865444	chr19:51727962	Sum eosinophil basophil counts	Astle W	27863252	European
rs3865444	chr19:51727962	Sum neutrophil eosinophil counts	Astle W	27863252	European
rs3865444	chr19:51727962	White blood cell count	Astle W	27863252	European
rs3865444	chr19:51727962	Alzheimers disease	Hollingworth P	21460840	European
rs3865444	chr19:51727962	Late onset Alzheimers disease	Naj AC	21460841	European
rs3865444	chr19:51727962	Alzheimers disease	IGAP	24162737	European
rs3865444	chr19:51727962	Alzheimers disease late onset	Naj AC	21460841	European
rs3865444	chr19:51727962	Alzheimers disease late onset	IGAP	24162737	European
rs3865444	chr19:51727962	Platelet count	Astle W	27863252	European

rs3865444	chr19:51727962	White blood cell count	Astle W	27863252	European
rs3865444	chr19:51727962	Alzheimer disease	Naj AC	21460841	European
rs1801274	chr1:161479745	Basophil count	Astle W	27863252	European
rs1801274	chr1:161479745	Basophil percentage of granulocytes	Astle W	27863252	European
rs1801274	chr1:161479745	Basophil percentage of white cells	Astle W	27863252	European
rs1801274	chr1:161479745	Inflammatory bowel disease	IBDGC	23128233	European
rs1801274	chr1:161479745	Kawasaki disease	Khor CC	22081228	Mixed
rs1801274	chr1:161479745	Kawasaki disease	Onouchi Y	22446962	East Asian
rs1801274	chr1:161479745	Systemic lupus erythematosus SLE females	Harley JB	18204446	European
rs1801274	chr1:161479745	Total cholesterol	Asselbergs	23063622	European
rs1801274	chr1:161479745	Total colectomy in ulcerative colitis	Asano K	19915573	East Asian
rs1801274	chr1:161479745	Ulcerative colitis	Asano K	19915573	East Asian
rs1801274	chr1:161479745	Ulcerative colitis	Anderson CA	21297633	European
rs1801274	chr1:161479745	Ulcerative colitis Smoking	Asano K	19915573	East Asian
rs1801274	chr1:161479745	Inflammatory bowel disease	IBDGC	26192919	European
rs1801274	chr1:161479745	Ulcerative colitis	IBDGC	23128233	European
rs1801274	chr1:161479745	Ulcerative colitis	IBDGC	26192919	European
rs1801274	chr1:161479745	Cerebrospinal fluid biomarker levels	Sasayama D	28031287	East Asian
rs1801274	chr1:161479745	Crohns disease	IBDGC	26192919	European
rs1801274	chr1:161479745	Inflammatory bowel disease	IBDGC	23128233	European
rs1801274	chr1:161479745	Inflammatory bowel disease	IBDGC	26192919	European
rs1801274	chr1:161479745	Inflammatory bowel disease	de Lange KM	28067908	Mixed
rs1801274	chr1:161479745	Kawasaki disease	Khor CC	22081228	Mixed
rs1801274	chr1:161479745	Kawasaki disease	Kwon YC	28886140	East Asian
rs1801274	chr1:161479745	Systemic lupus erythematosus	Armstrong DL	24871463	European

rs1801274	chr1:161479745	Systemic lupus erythematosus	Bentham J	26502338	European
rs1801274	chr1:161479745	Systemic lupus erythematosus	Lessard CJ	26663301	East Asian
rs1801274	chr1:161479745	Systemic lupus erythematosus	Morris DL	27399966	Mixed
rs1801274	chr1:161479745	Ulcerative colitis	Asano K	19915573	East Asian
rs1801274	chr1:161479745	Ulcerative colitis	Anderson CA	21297633	European
rs1801274	chr1:161479745	Ulcerative colitis	IBDGC	26192919	European
rs1801274	chr1:161479745	Ulcerative colitis	de Lange KM	28067908	Mixed
rs1801274	chr1:161479745	Parkinsons disease	Nalls M	25064009	European
rs1801274	chr1:161479745	Self-reported ulcerative colitis	Neale B	UKBB	European
rs1801274	chr1:161479745	Rheumatoid arthritis	Okada Y	24390342	European
rs1801274	chr1:161479745	Rheumatoid arthritis	Okada Y	24390342	Mixed
rs1801274	chr1:161479745	1c+mDC:%32+; CD1c+ mDC subset (CD32+)	Roederer M	25772697	European
rs1801274	chr1:161479745	CD161 on CD4mem; Memory CD4	Roederer M	25772697	European
rs1801274	chr1:161479745	CD27 on CD4 T; CD4 T	Roederer M	25772697	European
rs1801274	chr1:161479745	CD27 on CD8 T; CD8 T	Roederer M	25772697	European
rs1801274	chr1:161479745	CD27 on IgA+B; IgA+ B	Roederer M	25772697	European
rs1801274	chr1:161479745	CD27 on IgG+B; IgG+ B	Roederer M	25772697	European
rs1801274	chr1:161479745	CD32 on pDC; pDC	Roederer M	25772697	European
rs1801274	chr1:161479745	CD4mem:%preTh17 (1); pre-Th17 (CD161+PD1+CCR4+CXCR3-)	Roederer M	25772697	European
rs1801274	chr1:161479745	CD8:%TM-like; CD8 TM-like (CD27-CD28+CD127+CD244-CDRA+)	Roederer M	25772697	European
rs1801274	chr1:161479745	PD1 on CD4mem; Memory CD4	Roederer M	25772697	European
rs1801274	chr1:161479745	mDC:%32+; mDC subset (CD32+)	Roederer M	25772697	European
rs1801274	chr1:161479745	Colitis ulcerative	Asano K	19915573	East Asian

rs1801274	chr1:161479745	Colitis ulcerative	Anderson CA	21297633	European
rs1801274	chr1:161479745	Mucocutaneous lymph node syndrome	Khor CC	22081228	Mixed
rs58483275	chr19:51868443	Myositis	Neale B	UKBB	European
rs3865444	chr19:51727962	Eosinophil count	Astle W	27863252	European
rs3865444	chr19:51727962	Granulocyte count	Astle W	27863252	European
rs3865444	chr19:51727962	Lymphocyte count	Astle W	27863252	European
rs3865444	chr19:51727962	Monocyte count	Astle W	27863252	European
rs3865444	chr19:51727962	Myeloid white cell count	Astle W	27863252	European
rs3865444	chr19:51727962	Platelet count	Astle W	27863252	European
rs3865444	chr19:51727962	Plateletcrit	Astle W	27863252	European
rs3865444	chr19:51727962	Sum eosinophil basophil counts	Astle W	27863252	European
rs3865444	chr19:51727962	Sum neutrophil eosinophil counts	Astle W	27863252	European
rs3865444	chr19:51727962	White blood cell count	Astle W	27863252	European
rs3865444	chr19:51727962	Alzheimers disease	Hollingworth P	21460840	European
rs3865444	chr19:51727962	Late onset Alzheimers disease	Naj AC	21460841	European
rs3865444	chr19:51727962	Alzheimers disease	IGAP	24162737	European
rs3865444	chr19:51727962	Alzheimers disease late onset	Naj AC	21460841	European
rs3865444	chr19:51727962	Alzheimers disease late onset	IGAP	24162737	European
rs3865444	chr19:51727962	Platelet count	Astle W	27863252	European
rs3865444	chr19:51727962	White blood cell count	Astle W	27863252	European
rs3865444	chr19:51727962	Alzheimer disease	Naj AC	21460841	European
rs1801274	chr1:161479745	Basophil count	Astle W	27863252	European
rs1801274	chr1:161479745	Basophil percentage of granulocytes	Astle W	27863252	European
rs1801274	chr1:161479745	Basophil percentage of white cells	Astle W	27863252	European
rs1801274	chr1:161479745	Inflammatory bowel disease	IBDGC	23128233	European

rs1801274	chr1:161479745	Kawasaki disease	Khor CC	22081228	Mixed
rs1801274	chr1:161479745	Kawasaki disease	Onouchi Y	22446962	East Asian
rs1801274	chr1:161479745	Systemic lupus erythematosus SLE females	Harley JB	18204446	European
rs1801274	chr1:161479745	Total cholesterol	Asselbergs	23063622	European
rs1801274	chr1:161479745	Total colectomy in ulcerative colitis	Asano K	19915573	East Asian
rs1801274	chr1:161479745	Ulcerative colitis	Asano K	19915573	East Asian
rs1801274	chr1:161479745	Ulcerative colitis	Anderson CA	21297633	European
rs1801274	chr1:161479745	Ulcerative colitis Smoking	Asano K	19915573	East Asian
rs1801274	chr1:161479745	Inflammatory bowel disease	IBDGC	26192919	European
rs1801274	chr1:161479745	Ulcerative colitis	IBDGC	23128233	European
rs1801274	chr1:161479745	Ulcerative colitis	IBDGC	26192919	European
rs1801274	chr1:161479745	Cerebrospinal fluid biomarker levels	Sasayama D	28031287	East Asian
rs1801274	chr1:161479745	Crohns disease	IBDGC	26192919	European
rs1801274	chr1:161479745	Inflammatory bowel disease	IBDGC	23128233	European
rs1801274	chr1:161479745	Inflammatory bowel disease	IBDGC	26192919	European
rs1801274	chr1:161479745	Inflammatory bowel disease	de Lange KM	28067908	Mixed
rs1801274	chr1:161479745	Kawasaki disease	Khor CC	22081228	Mixed
rs1801274	chr1:161479745	Kawasaki disease	Kwon YC	28886140	East Asian
rs1801274	chr1:161479745	Systemic lupus erythematosus	Armstrong DL	24871463	European
rs1801274	chr1:161479745	Systemic lupus erythematosus	Bentham J	26502338	European
rs1801274	chr1:161479745	Systemic lupus erythematosus	Lessard CJ	26663301	East Asian
rs1801274	chr1:161479745	Systemic lupus erythematosus	Morris DL	27399966	Mixed
rs1801274	chr1:161479745	Ulcerative colitis	Asano K	19915573	East Asian
rs1801274	chr1:161479745	Ulcerative colitis	Anderson CA	21297633	European
rs1801274	chr1:161479745	Ulcerative colitis	IBDGC	26192919	European

rs1801274	chr1:161479745	Ulcerative colitis	de Lange KM	28067908	Mixed
rs1801274	chr1:161479745	Parkinsons disease	Nalls M	25064009	European
rs1801274	chr1:161479745	Self-reported ulcerative colitis	Neale B	UKBB	European
rs1801274	chr1:161479745	Rheumatoid arthritis	Okada Y	24390342	European
rs1801274	chr1:161479745	Rheumatoid arthritis	Okada Y	24390342	Mixed
rs1801274	chr1:161479745	1c+mDC:%32+; CD1c+ mDC subset (CD32+)	Roederer M	25772697	European
rs1801274	chr1:161479745	CD161 on CD4mem; Memory CD4	Roederer M	25772697	European
rs1801274	chr1:161479745	CD27 on CD4 T; CD4 T	Roederer M	25772697	European
rs1801274	chr1:161479745	CD27 on CD8 T; CD8 T	Roederer M	25772697	European
rs1801274	chr1:161479745	CD27 on IgA+B; IgA+ B	Roederer M	25772697	European
rs1801274	chr1:161479745	CD27 on IgG+B; IgG+ B	Roederer M	25772697	European
rs1801274	chr1:161479745	CD32 on pDC; pDC	Roederer M	25772697	European
rs1801274	chr1:161479745	CD4mem:%preTh17 (1); pre-Th17 (CD161+PD1+CCR4+CXCR3-)	Roederer M	25772697	European
rs1801274	chr1:161479745	CD8:%TM-like; CD8 TM-like (CD27-CD28+CD127+CD244-CDRA+)	Roederer M	25772697	European
rs1801274	chr1:161479745	PD1 on CD4mem; Memory CD4	Roederer M	25772697	European
rs1801274	chr1:161479745	mDC:%32+; mDC subset (CD32+)	Roederer M	25772697	European
rs1801274	chr1:161479745	Colitis ulcerative	Asano K	19915573	East Asian
rs1801274	chr1:161479745	Colitis ulcerative	Anderson CA	21297633	European
rs1801274	chr1:161479745	Mucocutaneous lymph node syndrome	Khor CC	22081228	Mixed
rs3865444	chr19:51727962	Eosinophil count	Astle W	27863252	European
rs3865444	chr19:51727962	Granulocyte count	Astle W	27863252	European
rs3865444	chr19:51727962	Lymphocyte count	Astle W	27863252	European
rs3865444	chr19:51727962	Monocyte count	Astle W	27863252	European

rs3865444	chr19:51727962	Myeloid white cell count	Astle W	27863252	European
rs3865444	chr19:51727962	Platelet count	Astle W	27863252	European
rs3865444	chr19:51727962	Plateletcrit	Astle W	27863252	European
rs3865444	chr19:51727962	Sum eosinophil basophil counts	Astle W	27863252	European
rs3865444	chr19:51727962	Sum neutrophil eosinophil counts	Astle W	27863252	European
rs3865444	chr19:51727962	White blood cell count	Astle W	27863252	European
rs3865444	chr19:51727962	Alzheimers disease	Hollingworth P	21460840	European
rs3865444	chr19:51727962	Late onset Alzheimers disease	Naj AC	21460841	European
rs3865444	chr19:51727962	Alzheimers disease	IGAP	24162737	European
rs3865444	chr19:51727962	Alzheimers disease late onset	Naj AC	21460841	European
rs3865444	chr19:51727962	Alzheimers disease late onset	IGAP	24162737	European
rs3865444	chr19:51727962	Platelet count	Astle W	27863252	European
rs3865444	chr19:51727962	White blood cell count	Astle W	27863252	European
rs3865444	chr19:51727962	Alzheimer disease	Naj AC	21460841	European
rs7245846	chr19:51731176	Lymphocyte count	Astle W	27863252	European
rs7245846	chr19:51731176	Platelet count	Astle W	27863252	European
rs7245846	chr19:51731176	Plateletcrit	Astle W	27863252	European
rs7245846	chr19:51731176	White blood cell count	Astle W	27863252	European
rs7245846	chr19:51731176	Alzheimers disease	IGAP	24162737	European
rs3865444	chr19:51727962	Eosinophil count	Astle W	27863252	European
rs3865444	chr19:51727962	Granulocyte count	Astle W	27863252	European
rs3865444	chr19:51727962	Lymphocyte count	Astle W	27863252	European
rs3865444	chr19:51727962	Monocyte count	Astle W	27863252	European
rs3865444	chr19:51727962	Myeloid white cell count	Astle W	27863252	European
rs3865444	chr19:51727962	Platelet count	Astle W	27863252	European

rs3865444	chr19:51727962	Plateletcrit	Astle W	27863252	European
rs3865444	chr19:51727962	Sum eosinophil basophil counts	Astle W	27863252	European
rs3865444	chr19:51727962	Sum neutrophil eosinophil counts	Astle W	27863252	European
rs3865444	chr19:51727962	White blood cell count	Astle W	27863252	European
rs3865444	chr19:51727962	Alzheimers disease	Hollingworth P	21460840	European
rs3865444	chr19:51727962	Late onset Alzheimers disease	Naj AC	21460841	European
rs3865444	chr19:51727962	Alzheimers disease	IGAP	24162737	European
rs3865444	chr19:51727962	Alzheimers disease late onset	Naj AC	21460841	European
rs3865444	chr19:51727962	Alzheimers disease late onset	IGAP	24162737	European
rs3865444	chr19:51727962	Platelet count	Astle W	27863252	European
rs3865444	chr19:51727962	White blood cell count	Astle W	27863252	European
rs3865444	chr19:51727962	Alzheimer disease	Naj AC	21460841	European
rs3865444	chr19:51727962	Eosinophil count	Astle W	27863252	European
rs3865444	chr19:51727962	Granulocyte count	Astle W	27863252	European
rs3865444	chr19:51727962	Lymphocyte count	Astle W	27863252	European
rs3865444	chr19:51727962	Monocyte count	Astle W	27863252	European
rs3865444	chr19:51727962	Myeloid white cell count	Astle W	27863252	European
rs3865444	chr19:51727962	Platelet count	Astle W	27863252	European
rs3865444	chr19:51727962	Plateletcrit	Astle W	27863252	European
rs3865444	chr19:51727962	Sum eosinophil basophil counts	Astle W	27863252	European
rs3865444	chr19:51727962	Sum neutrophil eosinophil counts	Astle W	27863252	European
rs3865444	chr19:51727962	White blood cell count	Astle W	27863252	European
rs3865444	chr19:51727962	Alzheimers disease	Hollingworth P	21460840	European
rs3865444	chr19:51727962	Late onset Alzheimers disease	Naj AC	21460841	European
rs3865444	chr19:51727962	Alzheimers disease	IGAP	24162737	European

rs3865444	chr19:51727962	Alzheimers disease late onset	Naj AC	21460841	European
rs3865444	chr19:51727962	Alzheimers disease late onset	IGAP	24162737	European
rs3865444	chr19:51727962	Platelet count	Astle W	27863252	European
rs3865444	chr19:51727962	White blood cell count	Astle W	27863252	European
rs3865444	chr19:51727962	Alzheimer disease	Naj AC	21460841	European
rs56043070	chr1:247719769	High light scatter percentage of red cells	Astle W	27863252	European
rs56043070	chr1:247719769	High light scatter reticulocyte count	Astle W	27863252	European
rs56043070	chr1:247719769	Lymphocyte count	Astle W	27863252	European
rs56043070	chr1:247719769	Mean corpuscular volume	Astle W	27863252	European
rs56043070	chr1:247719769	Mean platelet volume	Astle W	27863252	European
rs56043070	chr1:247719769	Platelet count	Astle W	27863252	European
rs56043070	chr1:247719769	Platelet distribution width	Astle W	27863252	European
rs56043070	chr1:247719769	Plateletcrit	Astle W	27863252	European
rs56043070	chr1:247719769	Reticulocyte count	Astle W	27863252	European
rs56043070	chr1:247719769	Reticulocyte fraction of red cells	Astle W	27863252	European
rs56043070	chr1:247719769	Mean platelet volume	Astle W	27863252	European
rs56043070	chr1:247719769	Platelet count	Astle W	27863252	European
rs56043070	chr1:247719769	Platelet distribution width	Astle W	27863252	European
rs56043070	chr1:247719769	Plateletcrit	Astle W	27863252	European
rs56043070	chr1:247719769	Reticulocyte count	Astle W	27863252	European
rs56043070	chr1:247719769	Reticulocyte fraction of red cells	Astle W	27863252	European
rs3865444	chr19:51727962	Eosinophil count	Astle W	27863252	European
rs3865444	chr19:51727962	Granulocyte count	Astle W	27863252	European
rs3865444	chr19:51727962	Lymphocyte count	Astle W	27863252	European
rs3865444	chr19:51727962	Monocyte count	Astle W	27863252	European

rs3865444	chr19:51727962	Myeloid white cell count	Astle W	27863252	European
rs3865444	chr19:51727962	Platelet count	Astle W	27863252	European
rs3865444	chr19:51727962	Plateletcrit	Astle W	27863252	European
rs3865444	chr19:51727962	Sum eosinophil basophil counts	Astle W	27863252	European
rs3865444	chr19:51727962	Sum neutrophil eosinophil counts	Astle W	27863252	European
rs3865444	chr19:51727962	White blood cell count	Astle W	27863252	European
rs3865444	chr19:51727962	Alzheimers disease	Hollingworth P	21460840	European
rs3865444	chr19:51727962	Late onset Alzheimers disease	Naj AC	21460841	European
rs3865444	chr19:51727962	Alzheimers disease	IGAP	24162737	European
rs3865444	chr19:51727962	Alzheimers disease late onset	Naj AC	21460841	European
rs3865444	chr19:51727962	Alzheimers disease late onset	IGAP	24162737	European
rs3865444	chr19:51727962	Platelet count	Astle W	27863252	European
rs3865444	chr19:51727962	White blood cell count	Astle W	27863252	European
rs3865444	chr19:51727962	Alzheimer disease	Naj AC	21460841	European
rs56043070	chr1:247719769	High light scatter percentage of red cells	Astle W	27863252	European
rs56043070	chr1:247719769	High light scatter reticulocyte count	Astle W	27863252	European
rs56043070	chr1:247719769	Lymphocyte count	Astle W	27863252	European
rs56043070	chr1:247719769	Mean corpuscular volume	Astle W	27863252	European
rs56043070	chr1:247719769	Mean platelet volume	Astle W	27863252	European
rs56043070	chr1:247719769	Platelet count	Astle W	27863252	European
rs56043070	chr1:247719769	Platelet distribution width	Astle W	27863252	European
rs56043070	chr1:247719769	Plateletcrit	Astle W	27863252	European
rs56043070	chr1:247719769	Reticulocyte count	Astle W	27863252	European
rs56043070	chr1:247719769	Reticulocyte fraction of red cells	Astle W	27863252	European
rs56043070	chr1:247719769	Mean platelet volume	Astle W	27863252	European

rs56043070	chr1:247719769	Platelet count	Astle W	27863252	European
rs56043070	chr1:247719769	Platelet distribution width	Astle W	27863252	European
rs56043070	chr1:247719769	Plateletcrit	Astle W	27863252	European
rs56043070	chr1:247719769	Reticulocyte count	Astle W	27863252	European
rs56043070	chr1:247719769	Reticulocyte fraction of red cells	Astle W	27863252	European
rs58483275	chr19:51868443	Myositis	Neale B	UKBB	European
rs7245846	chr19:51731176	Lymphocyte count	Astle W	27863252	European
rs7245846	chr19:51731176	Platelet count	Astle W	27863252	European
rs7245846	chr19:51731176	Plateletcrit	Astle W	27863252	European
rs7245846	chr19:51731176	White blood cell count	Astle W	27863252	European
rs7245846	chr19:51731176	Alzheimers disease	IGAP	24162737	European
rs7245846	chr19:51731176	Lymphocyte count	Astle W	27863252	European
rs7245846	chr19:51731176	Platelet count	Astle W	27863252	European
rs7245846	chr19:51731176	Plateletcrit	Astle W	27863252	European
rs7245846	chr19:51731176	White blood cell count	Astle W	27863252	European
rs7245846	chr19:51731176	Alzheimers disease	IGAP	24162737	European
rs58483275	chr19:51868443	Myositis	Neale B	UKBB	European
rs3865444	chr19:51727962	Eosinophil count	Astle W	27863252	European
rs3865444	chr19:51727962	Granulocyte count	Astle W	27863252	European
rs3865444	chr19:51727962	Lymphocyte count	Astle W	27863252	European
rs3865444	chr19:51727962	Monocyte count	Astle W	27863252	European
rs3865444	chr19:51727962	Myeloid white cell count	Astle W	27863252	European
rs3865444	chr19:51727962	Platelet count	Astle W	27863252	European
rs3865444	chr19:51727962	Plateletcrit	Astle W	27863252	European
rs3865444	chr19:51727962	Sum eosinophil basophil counts	Astle W	27863252	European

rs3865444	chr19:51727962	Sum neutrophil eosinophil counts	Astle W	27863252	European
rs3865444	chr19:51727962	White blood cell count	Astle W	27863252	European
rs3865444	chr19:51727962	Alzheimers disease	Hollingworth P	21460840	European
rs3865444	chr19:51727962	Late onset Alzheimers disease	Naj AC	21460841	European
rs3865444	chr19:51727962	Alzheimers disease	IGAP	24162737	European
rs3865444	chr19:51727962	Alzheimers disease late onset	Naj AC	21460841	European
rs3865444	chr19:51727962	Alzheimers disease late onset	IGAP	24162737	European
rs3865444	chr19:51727962	Platelet count	Astle W	27863252	European
rs3865444	chr19:51727962	White blood cell count	Astle W	27863252	European
rs3865444	chr19:51727962	Alzheimer disease	Naj AC	21460841	European
rs3865444	chr19:51727962	Eosinophil count	Astle W	27863252	European
rs3865444	chr19:51727962	Granulocyte count	Astle W	27863252	European
rs3865444	chr19:51727962	Lymphocyte count	Astle W	27863252	European
rs3865444	chr19:51727962	Monocyte count	Astle W	27863252	European
rs3865444	chr19:51727962	Myeloid white cell count	Astle W	27863252	European
rs3865444	chr19:51727962	Platelet count	Astle W	27863252	European
rs3865444	chr19:51727962	Plateletcrit	Astle W	27863252	European
rs3865444	chr19:51727962	Sum eosinophil basophil counts	Astle W	27863252	European
rs3865444	chr19:51727962	Sum neutrophil eosinophil counts	Astle W	27863252	European
rs3865444	chr19:51727962	White blood cell count	Astle W	27863252	European
rs3865444	chr19:51727962	Alzheimers disease	Hollingworth P	21460840	European
rs3865444	chr19:51727962	Late onset Alzheimers disease	Naj AC	21460841	European
rs3865444	chr19:51727962	Alzheimers disease	IGAP	24162737	European
rs3865444	chr19:51727962	Alzheimers disease late onset	Naj AC	21460841	European
rs3865444	chr19:51727962	Alzheimers disease late onset	IGAP	24162737	European

rs3865444	chr19:51727962	Platelet count	Astle W	27863252	European
rs3865444	chr19:51727962	White blood cell count	Astle W	27863252	European
rs3865444	chr19:51727962	Alzheimer disease	Naj AC	21460841	European
rs149007767	chr7:50370254	Basophil count	Astle W	27863252	European
rs149007767	chr7:50370254	Basophil percentage of white cells	Astle W	27863252	European
rs149007767	chr7:50370254	Granulocyte percentage of myeloid white cells	Astle W	27863252	European
rs149007767	chr7:50370254	Monocyte count	Astle W	27863252	European
rs149007767	chr7:50370254	Monocyte percentage of white cells	Astle W	27863252	European
rs149007767	chr7:50370254	Neutrophil percentage of white cells	Astle W	27863252	European
rs149007767	chr7:50370254	Granulocyte percentage of myeloid white cells	Astle W	27863252	European
rs149007767	chr7:50370254	Monocyte count	Astle W	27863252	European
rs149007767	chr7:50370254	Monocyte percentage of white cells	Astle W	27863252	European
rs149007767	chr7:50370254	Neutrophil percentage of white cells	Astle W	27863252	European
rs10098310	chr8:130613614	Basophil percentage of granulocytes	Astle W	27863252	European
rs10098310	chr8:130613614	Basophil percentage of white cells	Astle W	27863252	European
rs10098310	chr8:130613614	Eosinophil count	Astle W	27863252	European
rs10098310	chr8:130613614	Eosinophil percentage of granulocytes	Astle W	27863252	European
rs10098310	chr8:130613614	Eosinophil percentage of white cells	Astle W	27863252	European
rs10098310	chr8:130613614	Granulocyte count	Astle W	27863252	European
rs10098310	chr8:130613614	Granulocyte percentage of myeloid white cells	Astle W	27863252	European
rs10098310	chr8:130613614	Immature fraction of reticulocytes	Astle W	27863252	European
rs10098310	chr8:130613614	Lymphocyte percentage of white cells	Astle W	27863252	European
rs10098310	chr8:130613614	Mean corpuscular hemoglobin	Astle W	27863252	European
rs10098310	chr8:130613614	Mean corpuscular volume	Astle W	27863252	European
rs10098310	chr8:130613614	Monocyte count	Astle W	27863252	European

rs10098310	chr8:130613614	Monocyte percentage of white cells	Astle W	27863252	European
rs10098310	chr8:130613614	Myeloid white cell count	Astle W	27863252	European
rs10098310	chr8:130613614	Neutrophil count	Astle W	27863252	European
rs10098310	chr8:130613614	Neutrophil percentage of granulocytes	Astle W	27863252	European
rs10098310	chr8:130613614	Neutrophil percentage of white cells	Astle W	27863252	European
rs10098310	chr8:130613614	Red blood cell count	Astle W	27863252	European
rs10098310	chr8:130613614	Sum basophil neutrophil counts	Astle W	27863252	European
rs10098310	chr8:130613614	Sum eosinophil basophil counts	Astle W	27863252	European
rs10098310	chr8:130613614	Sum neutrophil eosinophil counts	Astle W	27863252	European
rs10098310	chr8:130613614	White blood cell count	Astle W	27863252	European
rs10098310	chr8:130613614	Inflammatory bowel disease	IBDGC	26192919	European
rs10098310	chr8:130613614	White blood cell count	Nalls MA	21738480	European
rs2239630	chr14:23589349	Basophil count	Astle W	27863252	European
rs2239630	chr14:23589349	Basophil percentage of granulocytes	Astle W	27863252	European
rs2239630	chr14:23589349	Basophil percentage of white cells	Astle W	27863252	European
rs2239630	chr14:23589349	Eosinophil count	Astle W	27863252	European
rs2239630	chr14:23589349	Eosinophil percentage of granulocytes	Astle W	27863252	European
rs2239630	chr14:23589349	Eosinophil percentage of white cells	Astle W	27863252	European
rs2239630	chr14:23589349	Granulocyte percentage of myeloid white cells	Astle W	27863252	European
rs2239630	chr14:23589349	Monocyte count	Astle W	27863252	European
rs2239630	chr14:23589349	Monocyte percentage of white cells	Astle W	27863252	European
rs2239630	chr14:23589349	Neutrophil percentage of granulocytes	Astle W	27863252	European
rs2239630	chr14:23589349	Neutrophil percentage of white cells	Astle W	27863252	European
rs2239630	chr14:23589349	Sum eosinophil basophil counts	Astle W	27863252	European
rs2239630	chr14:23589349	Granulocyte percentage of myeloid white cells	Astle W	27863252	European

rs2239630	chr14:23589349	Monocyte count	Astle W	27863252	European
rs2239630	chr14:23589349	Monocyte percentage of white cells	Astle W	27863252	European
rs2239630	chr14:23589349	Neutrophil percentage of granulocytes	Astle W	27863252	European
rs2239630	chr14:23589349	Neutrophil percentage of white cells	Astle W	27863252	European
rs2239630	chr14:23589349	Sum eosinophil basophil counts	Astle W	27863252	European
rs62115999	chr19:51761614	Self-reported ureteric obstruction or hydronephrosis	Neale B	UKBB	European
rs1710398	chr19:51726911	Platelet count	Astle W	27863252	European
rs1710398	chr19:51726911	Plateletcrit	Astle W	27863252	European
rs1710398	chr19:51726911	White blood cell count	Astle W	27863252	European
rs12459419	chr19:51728477	Eosinophil count	Astle W	27863252	European
rs12459419	chr19:51728477	Granulocyte count	Astle W	27863252	European
rs12459419	chr19:51728477	Lymphocyte count	Astle W	27863252	European
rs12459419	chr19:51728477	Monocyte count	Astle W	27863252	European
rs12459419	chr19:51728477	Myeloid white cell count	Astle W	27863252	European
rs12459419	chr19:51728477	Platelet count	Astle W	27863252	European
rs12459419	chr19:51728477	Plateletcrit	Astle W	27863252	European
rs12459419	chr19:51728477	Sum eosinophil basophil counts	Astle W	27863252	European
rs12459419	chr19:51728477	Sum neutrophil eosinophil counts	Astle W	27863252	European
rs12459419	chr19:51728477	White blood cell count	Astle W	27863252	European
rs12459419	chr19:51728477	Alzheimers disease	IGAP	24162737	European
rs12459419	chr19:51728477	Blood protein levels	Suhre K	28240269	European
rs12459419	chr19:51728477	Plateletcrit	Astle W	27863252	European

Supplementary table 4: Sensitivity analysis results for the bidirectional 2-sample MR.

AD as outcome	exposure	method	nsnp	pval	or	or_lci95	or_uci95

	blood gene expression of CD33	MR Egger	5	0.059	1.206	1.066	1.365
	blood gene expression of CD33	Weighted median	5	8.32E-05	1.162	1.078	1.252
	blood gene expression of CD33	IVW	5	3.25E-05	1.156	1.080	1.238
	blood gene expression of CD33	Simple mode	5	0.049	1.212	1.059	1.386
	blood gene expression of CD33	Weighted mode	5	0.019	1.166	1.078	1.261
	Serum protein level of CD33	IVW	2	0.002	1.084	1.031	1.139
	CD33 on CD14+ monocyte	MR Egger	5	0.088	1.017	1.082	1.151
	CD33 on CD14+ monocyte	Weighted median	5	2.41E-06	1.038	1.067	1.096
	CD33 on CD14+ monocyte	IVW	5	0.001	1.024	1.061	1.100
	CD33 on CD14+ monocyte	Simple mode	5	0.854	0.912	0.992	1.079
	CD33 on CD14+ monocyte	Weighted mode	5	0.007	1.045	1.073	1.102
	CD33 on CD66b++ myeloid cell	MR Egger	4	0.042	1.079	1.139	1.203
	CD33 on CD66b++ myeloid cell	Weighted median	4	2.75E-05	1.037	1.070	1.105
	CD33 on CD66b++ myeloid cell	IVW	4	0.022	1.009	1.065	1.124
	CD33 on CD66b++ myeloid cell	Simple mode	4	0.824	0.860	0.983	1.125
	CD33 on CD66b++ myeloid cell	Weighted mode	4	0.015	1.052	1.086	1.122
	CD33 on CD33dim HLA DR+ CD11b+	MR Egger	6	0.032	1.035	1.092	1.152
	CD33 on CD33dim HLA DR+ CD11b+	Weighted median	6	3.01E-05	1.030	1.058	1.086
	CD33 on CD33dim HLA DR+ CD11b+	IVW	6	0.006	1.016	1.055	1.095
	CD33 on CD33dim HLA DR+ CD11b+	Simple mode	6	0.638	0.873	0.973	1.084
	CD33 on CD33dim HLA DR+ CD11b+	Weighted mode	6	0.004	1.044	1.074	1.104
	CD33 on CD33dim HLA DR+ CD11b-	MR Egger	5	0.074	1.024	1.093	1.165
	CD33 on CD33dim HLA DR+ CD11b-	Weighted median	5	1.08E-04	1.028	1.057	1.088
	CD33 on CD33dim HLA DR+ CD11b-	IVW	5	0.015	1.011	1.055	1.102
	CD33 on CD33dim HLA DR+ CD11b-	Simple mode	5	0.587	0.850	0.963	1.091
	CD33 on CD33dim HLA DR+ CD11b-	Weighted mode	5	0.007	1.045	1.075	1.105

	CD33 on basophil	MR Egger	7	0.013	1.053	1.113	1.176
	CD34 on basophil	Weighted median	7	1.20E-04	1.025	1.052	1.080
	CD35 on basophil	IVW	7	0.009	1.011	1.046	1.082
	CD36 on basophil	Simple mode	7	0.424	0.844	0.950	1.069
	CD37 on basophil	Weighted mode	7	0.007	1.029	1.059	1.089
	CD33 on CD33dim HLA DR-	MR Egger	6	0.031	1.044	1.113	1.188
	CD33 on CD33dim HLA DR-	Weighted median	6	3.19E-07	1.043	1.071	1.100
	CD33 on CD33dim HLA DR-	IVW	6	0.006	1.016	1.057	1.100
	CD33 on CD33dim HLA DR-	Simple mode	6	0.431	0.815	0.940	1.083
	CD33 on CD33dim HLA DR-	Weighted mode	6	0.003	1.047	1.074	1.102
	CD33 on Immature Myeloid-Derived Suppressor Cells	MR Egger	6	0.023	1.048	1.108	1.172
	CD33 on Immature Myeloid-Derived Suppressor Cells	Weighted median	6	4.72E-05	1.029	1.058	1.087
	CD33 on Immature Myeloid-Derived Suppressor Cells	IVW	6	0.001	1.020	1.052	1.086
	CD33 on Immature Myeloid-Derived Suppressor Cells	Simple mode	6	0.504	0.879	0.966	1.062
	CD33 on Immature Myeloid-Derived Suppressor Cells	Weighted mode	6	0.011	1.030	1.060	1.092
	CD33 on Granulocytic Myeloid-Derived Suppressor Cells	MR Egger	3	0.228	1.034	1.131	1.238
	CD33 on Granulocytic Myeloid-Derived Suppressor Cells	Weighted median	3	2.55E-04	1.032	1.071	1.111
	CD33 on Granulocytic Myeloid-Derived Suppressor Cells	IVW	3	0.038	1.004	1.065	1.130
	CD33 on Granulocytic Myeloid-Derived Suppressor Cells	Simple mode	3	0.705	0.906	1.029	1.168
	CD33 on Granulocytic Myeloid-Derived Suppressor Cells	Weighted mode	3	0.063	1.036	1.077	1.119
	CD33 on CD33+ HLA DR+	MR Egger	3	0.133	1.063	1.110	1.158
	CD33 on CD33+ HLA DR+	Weighted median	3	1.55E-05	1.031	1.058	1.086
	CD33 on CD33+ HLA DR+	IVW	3	0.026	1.007	1.056	1.109
	CD33 on CD33+ HLA DR+	Simple mode	3	0.593	0.842	0.959	1.093
	CD33 on CD33+ HLA DR+	Weighted mode	3	0.041	1.038	1.066	1.094
	CD33 on CD33+ HLA DR+ CD14dim	MR Egger	3	0.133	1.063	1.109	1.158

	CD33 on CD33+ HLA DR+ CD14dim	Weighted median	3	3.37E-06	1.034	1.059	1.085
	CD33 on CD33+ HLA DR+ CD14dim	IVW	3	0.024	1.007	1.057	1.109
	CD33 on CD33+ HLA DR+ CD14dim	Simple mode	3	0.580	0.843	0.958	1.089
	CD33 on CD33+ HLA DR+ CD14dim	Weighted mode	3	0.036	1.041	1.067	1.094
	CD33 on CD33+ HLA DR+ CD14-	MR Egger	3	0.133	1.062	1.109	1.158
	CD33 on CD33+ HLA DR+ CD14-	Weighted median	3	1.11E-05	1.032	1.058	1.085
	CD33 on CD33+ HLA DR+ CD14-	IVW	3	0.027	1.006	1.056	1.108
	CD33 on CD33+ HLA DR+ CD14-	Simple mode	3	0.606	0.840	0.960	1.096
	CD33 on CD33+ HLA DR+ CD14-	Weighted mode	3	0.038	1.039	1.066	1.093
	CD33 on Monocytic Myeloid-Derived Suppressor Cells	MR Egger	3	0.156	1.057	1.116	1.177
	CD33 on Monocytic Myeloid-Derived Suppressor Cells	Weighted median	3	2.17E-05	1.032	1.060	1.089
	CD33 on Monocytic Myeloid-Derived Suppressor Cells	IVW	3	0.033	1.005	1.059	1.116
	CD33 on Monocytic Myeloid-Derived Suppressor Cells	Simple mode	3	0.550	0.834	0.953	1.088
	CD33 on Monocytic Myeloid-Derived Suppressor Cells	Weighted mode	3	0.039	1.043	1.072	1.102
AD as exposure	outcome	method	nsnp	pval	or	or_lci95	or_uci95
	blood gene expression of CD33	MR Egger	6	0.501	1.051	0.921	1.200
	blood gene expression of CD33	Weighted median	6	0.068	1.074	0.995	1.160
	blood gene expression of CD33	IVW	6	0.014	1.082	1.016	1.153
	blood gene expression of CD33	Simple mode	6	0.179	1.095	0.977	1.226
	blood gene expression of CD33	Weighted mode	6	0.196	1.065	0.980	1.157
	Serum protein level of CD33	MR Egger	18	0.392	0.877	0.960	1.051
	Serum protein level of CD33	Weighted median	18	0.168	0.852	0.936	1.028
	Serum protein level of CD33	IVW	18	0.499	0.915	0.978	1.044
	Serum protein level of CD33	Simple mode	18	0.604	0.803	0.954	1.134
	Serum protein level of CD33	Weighted mode	18	0.128	0.839	0.924	1.018
	CD33 on CD14+ monocyte	MR Egger	17	0.129	1.173	0.966	1.424

CD33 on CD14+ monocyte	Weighted median	17	0.280	1.091	0.932	1.278
CD33 on CD14+ monocyte	IVW	17	0.325	1.068	0.937	1.218
CD33 on CD14+ monocyte	Simple mode	17	0.290	1.142	0.901	1.447
CD33 on CD14+ monocyte	Weighted mode	17	0.305	1.089	0.930	1.275
CD33 on CD66b++ myeloid cell	MR Egger	17	0.610	1.070	0.829	1.382
CD33 on CD66b++ myeloid cell	Weighted median	17	0.712	1.033	0.869	1.229
CD33 on CD66b++ myeloid cell	IVW	17	0.606	1.044	0.886	1.231
CD33 on CD66b++ myeloid cell	Simple mode	17	0.981	0.997	0.757	1.312
CD33 on CD66b++ myeloid cell	Weighted mode	17	0.907	1.011	0.848	1.204
CD33 on CD33dim HLA DR+ CD11b+	MR Egger	17	0.112	1.173	0.975	1.411
CD33 on CD33dim HLA DR+ CD11b+	Weighted median	17	0.185	1.115	0.949	1.310
CD33 on CD33dim HLA DR+ CD11b+	IVW	17	0.194	1.085	0.959	1.228
CD33 on CD33dim HLA DR+ CD11b+	Simple mode	17	0.388	1.113	0.879	1.408
CD33 on CD33dim HLA DR+ CD11b+	Weighted mode	17	0.228	1.101	0.947	1.280
CD33 on CD33dim HLA DR+ CD11b-	MR Egger	17	0.119	1.169	0.972	1.406
CD33 on CD33dim HLA DR+ CD11b-	Weighted median	17	0.071	1.151	0.988	1.340
CD33 on CD33dim HLA DR+ CD11b-	IVW	17	0.177	1.088	0.962	1.230
CD33 on CD33dim HLA DR+ CD11b-	Simple mode	17	0.061	1.259	1.006	1.574
CD33 on CD33dim HLA DR+ CD11b-	Weighted mode	17	0.107	1.144	0.980	1.336
CD33 on basophil	MR Egger	17	0.231	1.123	0.936	1.349
CD34 on basophil	Weighted median	17	0.443	1.067	0.904	1.261
CD35 on basophil	IVW	17	0.676	1.026	0.909	1.159
CD36 on basophil	Simple mode	17	0.319	1.134	0.893	1.440
CD37 on basophil	Weighted mode	17	0.525	1.061	0.888	1.266
CD33 on CD33dim HLA DR-	MR Egger	17	0.293	1.113	0.918	1.348
CD33 on CD33dim HLA DR-	Weighted median	17	0.689	1.034	0.877	1.220

CD33 on CD33dim HLA DR-	IVW	17	0.654	1.030	0.906	1.170
CD33 on CD33dim HLA DR-	Simple mode	17	0.418	1.123	0.854	1.476
CD33 on CD33dim HLA DR-	Weighted mode	17	0.664	1.040	0.874	1.237
CD33 on Immature Myeloid-Derived Suppressor Cells	MR Egger	17	0.424	1.080	0.899	1.299
CD33 on Immature Myeloid-Derived Suppressor Cells	Weighted median	17	0.955	1.005	0.846	1.193
CD33 on Immature Myeloid-Derived Suppressor Cells	IVW	17	0.702	1.024	0.907	1.156
CD33 on Immature Myeloid-Derived Suppressor Cells	Simple mode	17	0.366	1.131	0.873	1.465
CD33 on Immature Myeloid-Derived Suppressor Cells	Weighted mode	17	0.773	1.026	0.866	1.215
CD33 on Granulocytic Myeloid-Derived Suppressor Cells	MR Egger	17	0.438	1.087	0.885	1.335
CD33 on Granulocytic Myeloid-Derived Suppressor Cells	Weighted median	17	0.705	1.036	0.864	1.241
CD33 on Granulocytic Myeloid-Derived Suppressor Cells	IVW	17	0.755	1.022	0.892	1.171
CD33 on Granulocytic Myeloid-Derived Suppressor Cells	Simple mode	17	0.608	1.070	0.830	1.380
CD33 on Granulocytic Myeloid-Derived Suppressor Cells	Weighted mode	17	0.651	1.041	0.876	1.238
CD33 on CD33+ HLA DR+	MR Egger	17	0.247	1.153	0.914	1.454
CD33 on CD33+ HLA DR+	Weighted median	17	0.239	1.103	0.937	1.300
CD33 on CD33+ HLA DR+	IVW	17	0.473	1.058	0.907	1.233
CD33 on CD33+ HLA DR+	Simple mode	17	0.995	0.999	0.780	1.279
CD33 on CD33+ HLA DR+	Weighted mode	17	0.397	1.075	0.914	1.265
CD33 on CD33+ HLA DR+ CD14dim	MR Egger	17	0.180	1.178	0.938	1.480
CD33 on CD33+ HLA DR+ CD14dim	Weighted median	17	0.264	1.096	0.933	1.286
CD33 on CD33+ HLA DR+ CD14dim	IVW	17	0.377	1.071	0.920	1.247
CD33 on CD33+ HLA DR+ CD14dim	Simple mode	17	0.925	1.012	0.795	1.287
CD33 on CD33+ HLA DR+ CD14dim	Weighted mode	17	0.324	1.093	0.921	1.298
CD33 on CD33+ HLA DR+ CD14-	MR Egger	17	0.272	1.141	0.910	1.432
CD33 on CD33+ HLA DR+ CD14-	Weighted median	17	0.215	1.111	0.940	1.314
CD33 on CD33+ HLA DR+ CD14-	IVW	17	0.495	1.054	0.907	1.224

CD33 on CD33+ HLA DR+ CD14-	Simple mode	17	0.852	1.024	0.803	1.305
CD33 on CD33+ HLA DR+ CD14-	Weighted mode	17	0.418	1.070	0.912	1.256
CD33 on Monocytic Myeloid-Derived Suppressor Cells	MR Egger	17	0.113	1.186	0.972	1.448
CD33 on Monocytic Myeloid-Derived Suppressor Cells	Weighted median	17	0.387	1.077	0.910	1.275
CD33 on Monocytic Myeloid-Derived Suppressor Cells	IVW	17	0.771	1.022	0.885	1.179
CD33 on Monocytic Myeloid-Derived Suppressor Cells	Simple mode	17	0.994	0.999	0.776	1.286
CD33 on Monocytic Myeloid-Derived Suppressor Cells	Weighted mode	17	0.432	1.069	0.909	1.256

or: odds ratio; lci95: lower 95% confidential interval; uci95: upper95% confidential interval.

Supplementary table 5: IVs used in the reverse MR analyses(AD as exposure, CD33 as outcome).

exposure	samplesize	se	pval	beta	pos	SNP	EA	OA	eaf
AD	63926	0.0183	1.55E-16	-0.1508	rs679515	1:207750568	C	T	NA
AD	63926	0.0154	4.02E-28	0.1693	rs6733839	2:127892810	T	C	NA
AD	63926	0.0431	4.47E-12	0.298	rs114812713	6:41034000	C	G	NA
AD	63926	0.0166	5.80E-09	-0.0967	rs34665982	6:32560306	C	T	NA
AD	63926	0.0148	2.93E-08	-0.0821	rs9381563	6:47432637	T	C	NA
AD	63926	0.0182	1.56E-08	-0.1028	rs11767557	7:143109139	C	T	NA
AD	63926	0.0158	3.49E-17	0.1333	rs867230	8:27468503	A	C	NA
AD	63926	0.0153	8.34E-10	0.0936	rs73223431	8:27219987	T	C	NA
AD	63926	0.0154	4.64E-08	0.0841	rs11257242	10:11721119	G	C	NA
AD	63926	0.0149	1.19E-16	-0.1232	rs1582763	11:60021948	A	G	NA
AD	63926	0.0148	5.81E-16	0.1198	rs3851179	11:85868640	C	T	NA
AD	63926	0.0144	9.70E-11	0.0935	rs3740688	11:47380340	T	G	NA
AD	63926	0.0157	8.73E-09	-0.0906	rs12590654	14:92938855	A	G	NA
AD	63926	0.0811	2.27E-11	-0.5425	rs72654445	19:45417200	A	G	NA

AD	63926	0.0305	6.40E-53	-0.4673	rs7412	19:45412079	T	C	NA
AD	63926	0.0436	1.51E-103	0.942	rs1081105	19:45412955	C	A	NA
AD	63926	0.0169	2.56E-10	-0.1071	rs12151021	19:1050874	G	A	NA
AD	63926	0.0713	3.20E-11	-0.4735	rs111278137	19:45215081	A	G	NA
AD	63926	0.0366	1.00E-200	1.1354	rs147711004	19:45337918	A	G	NA
AD	63926	0.0851	6.43E-09	-0.4938	rs139136389	19:45427136	T	C	NA
AD	63926	0.0645	6.62E-18	0.5561	rs150685845	19:45675180	G	A	NA

EA: effect allele; OA: other allele; eaf: allele frequency of effect allele; NA: not available.

Supplementary table 6: Phenoscanncer search results of the IVs used in the reverse MR analyses (AD as exposure, CD33 as outcome).

snp	position	trait	study	pmid	ancestry
rs679515	chr1:207750568	Alzheimers disease	IGAP	24162737	European
rs679515	chr1:207750568	Alzheimers disease in APOE e4 carriers	Jun G	25778476	Unspecified
rs679515	chr1:207750568	Illnesses of mother: alzheimers disease or dementia	Neale B	UKBB	European
rs6733839	chr2:127892810	Alzheimers disease	IGAP	24162737	European
rs6733839	chr2:127892810	Alzheimers disease in APOE e4 carriers	Jun G	25778476	Unspecified
rs6733839	chr2:127892810	Alzheimers disease late onset	IGAP	24162737	European
rs6733839	chr2:127892810	Dementia and core Alzheimers disease neuropathologic changes	Beecham GW	25188341	Unspecified
rs6733839	chr2:127892810	Illnesses of father: alzheimers disease or dementia	Neale B	UKBB	European
rs6733839	chr2:127892810	Illnesses of mother: alzheimers disease or dementia	Neale B	UKBB	European
rs34665982	chr6:32560306	Hematocrit	Astle W	27863252	European
rs34665982	chr6:32560306	Hemoglobin concentration	Astle W	27863252	European
rs34665982	chr6:32560306	High light scatter percentage of red cells	Astle W	27863252	European
rs34665982	chr6:32560306	High light scatter reticulocyte count	Astle W	27863252	European

rs34665982	chr6:32560306	Lymphocyte count	Astle W	27863252	European
rs34665982	chr6:32560306	Mean corpuscular hemoglobin concentration	Astle W	27863252	European
rs34665982	chr6:32560306	Red blood cell count	Astle W	27863252	European
rs34665982	chr6:32560306	Reticulocyte count	Astle W	27863252	European
rs34665982	chr6:32560306	Reticulocyte fraction of red cells	Astle W	27863252	European
rs34665982	chr6:32560306	White blood cell count	Astle W	27863252	European
rs34665982	chr6:32560306	Inflammatory bowel disease	IBDGC	26192919	European
rs34665982	chr6:32560306	Ulcerative colitis	IBDGC	26192919	European
rs9381563	chr6:47432637	High light scatter percentage of red cells	Astle W	27863252	European
rs9381563	chr6:47432637	High light scatter reticulocyte count	Astle W	27863252	European
rs9381563	chr6:47432637	Immature fraction of reticulocytes	Astle W	27863252	European
rs9381563	chr6:47432637	Mean platelet volume	Astle W	27863252	European
rs9381563	chr6:47432637	Platelet count	Astle W	27863252	European
rs9381563	chr6:47432637	Platelet distribution width	Astle W	27863252	European
rs9381563	chr6:47432637	Red cell distribution width	Astle W	27863252	European
rs9381563	chr6:47432637	Reticulocyte count	Astle W	27863252	European
rs9381563	chr6:47432637	Reticulocyte fraction of red cells	Astle W	27863252	European
rs9381563	chr6:47432637	Late onset Alzheimers disease	Naj AC	21460841	European
rs9381563	chr6:47432637	Alzheimers disease	IGAP	24162737	European
rs9381563	chr6:47432637	Comparative height size at age 10	Neale B	UKBB	European
rs9381563	chr6:47432637	Height	Neale B	UKBB	European
rs11767557	chr7:143109139	Alzheimers disease	Hollingworth P	21460840	European
rs11767557	chr7:143109139	Late onset Alzheimers disease	Naj AC	21460841	European
rs11767557	chr7:143109139	Alzheimers disease	IGAP	24162737	European
rs11767557	chr7:143109139	Alzheimers disease late onset	Naj AC	21460841	European

rs11767557	chr7:143109139	Alzheimer disease	Naj AC	21460841	European
rs867230	chr8:27468503	Illnesses of mother: alzheimers disease or dementia	Neale B	UKBB	European
rs73223431	chr8:27219987	Alzheimers disease	IGAP	24162737	European
rs11257242	chr10:11721119	Alzheimers disease	IGAP	24162737	European
rs1582763	chr11:60021948	Granulocyte count	Astle W	27863252	European
rs1582763	chr11:60021948	Lymphocyte percentage of white cells	Astle W	27863252	European
rs1582763	chr11:60021948	Myeloid white cell count	Astle W	27863252	European
rs1582763	chr11:60021948	Neutrophil count	Astle W	27863252	European
rs1582763	chr11:60021948	Neutrophil percentage of white cells	Astle W	27863252	European
rs1582763	chr11:60021948	Sum basophil neutrophil counts	Astle W	27863252	European
rs1582763	chr11:60021948	Sum neutrophil eosinophil counts	Astle W	27863252	European
rs1582763	chr11:60021948	Late onset Alzheimers disease	Naj AC	21460841	European
rs1582763	chr11:60021948	Alzheimers disease	IGAP	24162737	European
rs1582763	chr11:60021948	Alzheimers disease in APOE e4 carriers	Jun G	25778476	Unspecified
rs1582763	chr11:60021948	Heel bone mineral density	Neale B	UKBB	European
rs1582763	chr11:60021948	Heel bone mineral density left	Neale B	UKBB	European
rs1582763	chr11:60021948	Heel bone mineral density right	Neale B	UKBB	European
rs3851179	chr11:85868640	High light scatter percentage of red cells	Astle W	27863252	European
rs3851179	chr11:85868640	High light scatter reticulocyte count	Astle W	27863252	European
rs3851179	chr11:85868640	Alzheimers disease	Harold D	19734902	European
rs3851179	chr11:85868640	Alzheimers disease	Seshadri S	20460622	European
rs3851179	chr11:85868640	Alzheimers disease	Furney SJ	21116278	European
rs3851179	chr11:85868640	Alzheimers disease	Hollingworth P	21460840	European
rs3851179	chr11:85868640	Late onset Alzheimers disease	Naj AC	21460841	European
rs3851179	chr11:85868640	Alzheimers disease	IGAP	24162737	European

rs3851179	chr11:85868640	Alzheimers disease	Harold D	19734902	European
rs3851179	chr11:85868640	Alzheimers disease in APOE e4 carriers	Jun G	25778476	Unspecified
rs3851179	chr11:85868640	Alzheimer disease	Harold D	19734902	European
rs3740688	chr11:47380340	Alzheimers disease	IGAP	24162737	European
rs3740688	chr11:47380340	Age at menarche	Neale B	UKBB	European
rs3740688	chr11:47380340	Arm fat mass left	Neale B	UKBB	European
rs3740688	chr11:47380340	Arm fat mass right	Neale B	UKBB	European
rs3740688	chr11:47380340	Arm fat percentage left	Neale B	UKBB	European
rs3740688	chr11:47380340	Arm fat percentage right	Neale B	UKBB	European
rs3740688	chr11:47380340	Body fat percentage	Neale B	UKBB	European
rs3740688	chr11:47380340	Body mass index	Neale B	UKBB	European
rs3740688	chr11:47380340	Comparative body size at age 10	Neale B	UKBB	European
rs3740688	chr11:47380340	Fluid intelligence score	Neale B	UKBB	European
rs3740688	chr11:47380340	Height	Neale B	UKBB	European
rs3740688	chr11:47380340	Hip circumference	Neale B	UKBB	European
rs3740688	chr11:47380340	Impedance of leg left	Neale B	UKBB	European
rs3740688	chr11:47380340	Impedance of leg right	Neale B	UKBB	European
rs3740688	chr11:47380340	Leg fat mass left	Neale B	UKBB	European
rs3740688	chr11:47380340	Leg fat mass right	Neale B	UKBB	European
rs3740688	chr11:47380340	Leg fat percentage left	Neale B	UKBB	European
rs3740688	chr11:47380340	Leg fat percentage right	Neale B	UKBB	European
rs3740688	chr11:47380340	Miserableness	Neale B	UKBB	European
rs3740688	chr11:47380340	Nervous feelings	Neale B	UKBB	European
rs3740688	chr11:47380340	Neuroticism score	Neale B	UKBB	European
rs3740688	chr11:47380340	Sitting height	Neale B	UKBB	European

rs3740688	chr11:47380340	Suffer from nerves	Neale B	UKBB	European
rs3740688	chr11:47380340	Trunk fat mass	Neale B	UKBB	European
rs3740688	chr11:47380340	Trunk fat percentage	Neale B	UKBB	European
rs3740688	chr11:47380340	Usual walking pace	Neale B	UKBB	European
rs3740688	chr11:47380340	Waist circumference	Neale B	UKBB	European
rs3740688	chr11:47380340	Weight	Neale B	UKBB	European
rs3740688	chr11:47380340	Whole body fat mass	Neale B	UKBB	European
rs12590654	chr14:92938855	Alzheimers disease	IGAP	24162737	European
rs7412	chr19:45412079	High light scatter percentage of red cells	Astle W	27863252	European
rs7412	chr19:45412079	High light scatter reticulocyte count	Astle W	27863252	European
rs7412	chr19:45412079	Immature fraction of reticulocytes	Astle W	27863252	European
rs7412	chr19:45412079	Plateletcrit	Astle W	27863252	European
rs7412	chr19:45412079	Red cell distribution width	Astle W	27863252	European
rs7412	chr19:45412079	Reticulocyte count	Astle W	27863252	European
rs7412	chr19:45412079	Reticulocyte fraction of red cells	Astle W	27863252	European
rs7412	chr19:45412079	Coronary artery disease	CARDIoGRAMplusC4D	26343387	Mixed
rs7412	chr19:45412079	Myocardial infarction	CARDIoGRAMplusC4D	26343387	Mixed
rs7412	chr19:45412079	Age-related macular degeneration	Fritsche LG	26691988	European
rs7412	chr19:45412079	High density lipoprotein	GLGC	24097068	European
rs7412	chr19:45412079	Low density lipoprotein	GLGC	24097068	European
rs7412	chr19:45412079	Total cholesterol	GLGC	24097068	European
rs7412	chr19:45412079	Triglycerides	GLGC	24097068	European
rs7412	chr19:45412079	APOB apolipoprotein B	Hopewell	23100282	European
rs7412	chr19:45412079	APOB apolipoprotein B response after 40mg daily simvastatin treatment	Hopewell	23100282	European
rs7412	chr19:45412079	LDL cholesterol	Smith EN	20838585	European

rs7412	chr19:45412079	LDL cholesterol	Asselbergs	23063622	European
rs7412	chr19:45412079	LDL cholesterol	Rasmussen Torvik LJ	23067351	African
rs7412	chr19:45412079	LDL cholesterol	Hopewell	23100282	European
rs7412	chr19:45412079	LDL cholesterol among a group where cancer diabetes hyperhypothyroidism and LDL altering medications were not present	Rasmussen Torvik LJ	23067351	African
rs7412	chr19:45412079	LDL cholesterol change with statins	Thompson	20031582	Mixed
rs7412	chr19:45412079	LDL cholesterol response after 40mg daily simvastatin treatment	Hopewell	23100282	European
rs7412	chr19:45412079	LDL cholesterol response to statins baseline LDL cholesterol	Chasman DI	22331829	European
rs7412	chr19:45412079	LDL cholesterol response to statins fractional change in LDL cholesterol	Chasman DI	22331829	European
rs7412	chr19:45412079	LDL cholesterol response to statins residuals of the measure of fractional change in LDL cholesterol	Chasman DI	22331829	European
rs7412	chr19:45412079	Late onset Alzheimers disease	Naj AC	20885792	Unspecified
rs7412	chr19:45412079	Late onset Alzheimers disease	Hu X	21390209	European
rs7412	chr19:45412079	Lipoprotein associated phospholipase A2 activity Lp2	Chu AY	23118302	European
rs7412	chr19:45412079	Total cholesterol	Asselbergs	23063622	European
rs7412	chr19:45412079	Alzheimers disease	IGAP	24162737	European
rs7412	chr19:45412079	Cholesterol total	Surakka I	25961943	European
rs7412	chr19:45412079	Cholesterol total	Nagy R	28270201	European
rs7412	chr19:45412079	Coronary artery disease	Nelson CP	28714975	Mixed
rs7412	chr19:45412079	Coronary artery disease	van der Harst P	29212778	Mixed
rs7412	chr19:45412079	Coronary artery disease	van der Harst P	29212778	Mixed
rs7412	chr19:45412079	Coronary artery disease	van der Harst P	29212778	Mixed
rs7412	chr19:45412079	HDL cholesterol	Nagy R	28270201	European
rs7412	chr19:45412079	High light scatter reticulocyte count	Astle W	27863252	European
rs7412	chr19:45412079	High light scatter reticulocyte percentage of red cells	Astle W	27863252	European

rs7412	chr19:45412079	Ideal cardiovascular health clinical and behavioural	Allen NB	27179730	European
rs7412	chr19:45412079	Immature fraction of reticulocytes	Astle W	27863252	European
rs7412	chr19:45412079	LDL cholesterol	Rasmussen Torvik LJ	23067351	African
rs7412	chr19:45412079	LDL cholesterol levels	Spracklen CN	28334899	East Asian
rs7412	chr19:45412079	LDL cholesterol levels	Zhu Y	28371326	East Asian
rs7412	chr19:45412079	Lipid metabolism phenotypes	Kettunen J	22286219	European
rs7412	chr19:45412079	Lipid traits	Wu Y	24023260	Filipino
rs7412	chr19:45412079	Lipid traits	Wu Y	24023260	Filipino
rs7412	chr19:45412079	Lipoprotein a levels	Mack S	28512139	European
rs7412	chr19:45412079	Lipoprotein associated phospholipase A2 activity change in response to darapladib treatment in cardiovascular disease	Yeo A	28753643	Mixed
rs7412	chr19:45412079	Lipoprotein phospholipase A2 activity in cardiovascular disease	Yeo A	28753643	Mixed
rs7412	chr19:45412079	Lipoproteina levels adjusted for apolipoproteina isoforms	Mack S	28512139	European
rs7412	chr19:45412079	Low density lipoprotein cholesterol	Southam L	28548082	Mixed
rs7412	chr19:45412079	Metabolite levels lipoprotein measures	Kettunen J	27005778	European
rs7412	chr19:45412079	Pulse pressure	Warren HR	28135244	European
rs7412	chr19:45412079	Red cell distribution width	Astle W	27863252	European
rs7412	chr19:45412079	Response to statin therapy LDL C	Chasman DI	22331829	European
rs7412	chr19:45412079	Reticulocyte count	Astle W	27863252	European
rs7412	chr19:45412079	Reticulocyte fraction of red cells	Astle W	27863252	European
rs7412	chr19:45412079	Total cholesterol levels	Spracklen CN	28334899	East Asian
rs7412	chr19:45412079	Total cholesterol levels	Southam L	28548082	Mixed
rs7412	chr19:45412079	Arm fat mass left	Neale B	UKBB	European
rs7412	chr19:45412079	Arm fat mass right	Neale B	UKBB	European
rs7412	chr19:45412079	Arm fat percentage left	Neale B	UKBB	European

rs7412	chr19:45412079	Arm fat percentage right	Neale B	UKBB	European
rs7412	chr19:45412079	Blood clot in the leg	Neale B	UKBB	European
rs7412	chr19:45412079	Body fat percentage	Neale B	UKBB	European
rs7412	chr19:45412079	Body mass index	Neale B	UKBB	European
rs7412	chr19:45412079	Chronic ischaemic heart disease	Neale B	UKBB	European
rs7412	chr19:45412079	Fathers age at death	Neale B	UKBB	European
rs7412	chr19:45412079	Hip circumference	Neale B	UKBB	European
rs7412	chr19:45412079	Illnesses of father: alzheimers disease or dementia	Neale B	UKBB	European
rs7412	chr19:45412079	Illnesses of father: heart disease	Neale B	UKBB	European
rs7412	chr19:45412079	Illnesses of mother: alzheimers disease or dementia	Neale B	UKBB	European
rs7412	chr19:45412079	Illnesses of mother: none of the above, group 1	Neale B	UKBB	European
rs7412	chr19:45412079	Leg fat mass left	Neale B	UKBB	European
rs7412	chr19:45412079	Leg fat mass right	Neale B	UKBB	European
rs7412	chr19:45412079	Leg fat percentage right	Neale B	UKBB	European
rs7412	chr19:45412079	Medication for cholesterol, blood pressure or diabetes: cholesterol lowering medication	Neale B	UKBB	European
rs7412	chr19:45412079	Medication for cholesterol, blood pressure or diabetes: none of the above	Neale B	UKBB	European
rs7412	chr19:45412079	Medication for pain relief, constipation, heartburn: aspirin	Neale B	UKBB	European
rs7412	chr19:45412079	No treatment with medication for cholesterol, blood pressure, diabetes, or take exogenous hormones	Neale B	UKBB	European
rs7412	chr19:45412079	Number of self-reported non-cancer illnesses	Neale B	UKBB	European
rs7412	chr19:45412079	Number of treatments or medications taken	Neale B	UKBB	European
rs7412	chr19:45412079	Pulse rate	Neale B	UKBB	European
rs7412	chr19:45412079	Pulse rate	Neale B	UKBB	European
rs7412	chr19:45412079	Self-reported angina	Neale B	UKBB	European

rs7412	chr19:45412079	Self-reported deep venous thrombosis	Neale B	UKBB	European
rs7412	chr19:45412079	Self-reported heart attack or myocardial infarction	Neale B	UKBB	European
rs7412	chr19:45412079	Self-reported high cholesterol	Neale B	UKBB	European
rs7412	chr19:45412079	Self-reported hypertension	Neale B	UKBB	European
rs7412	chr19:45412079	Treatment with aspirin	Neale B	UKBB	European
rs7412	chr19:45412079	Treatment with atorvastatin	Neale B	UKBB	European
rs7412	chr19:45412079	Treatment with cholesterol lowering medication	Neale B	UKBB	European
rs7412	chr19:45412079	Treatment with ezetimibe	Neale B	UKBB	European
rs7412	chr19:45412079	Treatment with lipitor 10mg tablet	Neale B	UKBB	European
rs7412	chr19:45412079	Treatment with rosuvastatin	Neale B	UKBB	European
rs7412	chr19:45412079	Treatment with simvastatin	Neale B	UKBB	European
rs7412	chr19:45412079	Trunk fat mass	Neale B	UKBB	European
rs7412	chr19:45412079	Trunk fat percentage	Neale B	UKBB	European
rs7412	chr19:45412079	Vascular or heart problems diagnosed by doctor: angina	Neale B	UKBB	European
rs7412	chr19:45412079	Vascular or heart problems diagnosed by doctor: heart attack	Neale B	UKBB	European
rs7412	chr19:45412079	Vascular or heart problems diagnosed by doctor: high blood pressure	Neale B	UKBB	European
rs7412	chr19:45412079	Vascular or heart problems diagnosed by doctor: none of the above	Neale B	UKBB	European
rs7412	chr19:45412079	Waist circumference	Neale B	UKBB	European
rs7412	chr19:45412079	Weight	Neale B	UKBB	European
rs7412	chr19:45412079	Whole body fat mass	Neale B	UKBB	European
rs7412	chr19:45412079	Coronary artery disease	Nelson CP	28714975	Mixed
rs7412	chr19:45412079	Low density lipoprotein	Prins B	28887542	European
rs7412	chr19:45412079	Triglycerides	Prins B	28887542	European
rs7412	chr19:45412079	Total cholesterol	Prins B	28887542	European
rs7412	chr19:45412079	High density lipoprotein	Spracklen CN	28334899	East Asian

rs7412	chr19:45412079	Low density lipoprotein	Spracklen CN	28334899	East Asian
rs7412	chr19:45412079	Total cholesterol	Spracklen CN	28334899	East Asian
rs7412	chr19:45412079	Triglycerides	Spracklen CN	28334899	East Asian
rs7412	chr19:45412079	Cholesterol ldl	Chasman DI	22331829	European
rs7412	chr19:45412079	Lipid metabolism	Kettunen J	22286219	European
rs7412	chr19:45412079	Coronary artery disease	van der Harst P	29212778	Mixed
rs7412	chr19:45412079	Coronary artery disease	van der Harst P	29212778	Mixed
rs1081105	chr19:45412955	Red cell distribution width	Astle W	27863252	European
rs1081105	chr19:45412955	Age-related macular degeneration	Fritsche LG	26691988	European
rs1081105	chr19:45412955	Alzheimers disease	IGAP	24162737	European
rs1081105	chr19:45412955	Body fat percentage	Neale B	UKBB	European
rs1081105	chr19:45412955	Cause of death: unspecified dementia	Neale B	UKBB	European
rs1081105	chr19:45412955	Illnesses of father: alzheimers disease or dementia	Neale B	UKBB	European
rs1081105	chr19:45412955	Illnesses of mother: alzheimers disease or dementia	Neale B	UKBB	European
rs1081105	chr19:45412955	Leg fat percentage left	Neale B	UKBB	European
rs1081105	chr19:45412955	Leg fat percentage right	Neale B	UKBB	European
rs1081105	chr19:45412955	Medication for cholesterol, blood pressure or diabetes: cholesterol lowering medication	Neale B	UKBB	European
rs1081105	chr19:45412955	Self-reported high cholesterol	Neale B	UKBB	European
rs1081105	chr19:45412955	Treatment with atorvastatin	Neale B	UKBB	European
rs1081105	chr19:45412955	Treatment with cholesterol lowering medication	Neale B	UKBB	European
rs1081105	chr19:45412955	Treatment with ezetimibe	Neale B	UKBB	European
rs1081105	chr19:45412955	Treatment with lipitor 10mg tablet	Neale B	UKBB	European
rs1081105	chr19:45412955	Treatment with simvastatin	Neale B	UKBB	European
rs1081105	chr19:45412955	Waist circumference	Neale B	UKBB	European

rs1081105	chr19:45412955	C-reactive protein	Prins B	28887542	European
rs12151021	chr19:1050874	Lymphocyte count	Astle W	27863252	European
rs12151021	chr19:1050874	Mean corpuscular hemoglobin concentration	Astle W	27863252	European
rs12151021	chr19:1050874	Red cell distribution width	Astle W	27863252	European
rs12151021	chr19:1050874	Alzheimers disease	IGAP	24162737	European
rs147711004	chr19:45337918	Illnesses of father: alzheimers disease or dementia	Neale B	UKBB	European
rs147711004	chr19:45337918	Illnesses of mother: alzheimers disease or dementia	Neale B	UKBB	European
rs147711004	chr19:45337918	Illnesses of mother: none of the above, group 1	Neale B	UKBB	European
rs147711004	chr19:45337918	Illnesses of siblings: alzheimers disease or dementia	Neale B	UKBB	European
rs147711004	chr19:45337918	Leg fat mass left	Neale B	UKBB	European
rs147711004	chr19:45337918	Leg fat percentage left	Neale B	UKBB	European
rs147711004	chr19:45337918	Medication for cholesterol, blood pressure or diabetes: cholesterol lowering medication	Neale B	UKBB	European
rs147711004	chr19:45337918	Self-reported high cholesterol	Neale B	UKBB	European
rs147711004	chr19:45337918	Treatment with simvastatin	Neale B	UKBB	European
rs147711004	chr19:45337918	Waist circumference	Neale B	UKBB	European
rs147711004	chr19:45337918	Low density lipoprotein	Prins B	28887542	European
rs147711004	chr19:45337918	Coronary artery disease	van der Harst P	29212778	Mixed
rs147711004	chr19:45337918	Coronary artery disease	van der Harst P	29212778	Mixed
rs150685845	chr19:45675180	Alzheimers disease	IGAP	24162737	European
rs150685845	chr19:45675180	Illnesses of father: alzheimers disease or dementia	Neale B	UKBB	European
rs150685845	chr19:45675180	Illnesses of mother: alzheimers disease or dementia	Neale B	UKBB	European
